# A multiscale natural community and species-level vulnerability assessment of the Gulf Coast, USA

**DOI:** 10.1371/journal.pone.0199844

**Published:** 2018-06-29

**Authors:** Joshua Steven Reece, Amanda Watson, Patricia Soupy Dalyander, Cynthia Kallio Edwards, Laura Geselbracht, Megan K. LaPeyre, Blair E. Tirpak, John M. Tirpak, Mark Woodrey

**Affiliations:** 1 Department of Biology, California State University at Fresno, Fresno, California, United States of America; 2 Mississippi State University, Starkville, Mississippi, United States of America; 3 U.S. Geological Survey, St. Petersburg Coastal and Marine Science Center, St. Petersburg, Florida, United States of America; 4 Gulf Coast Prairie Landscape Conservation Cooperative, Wetland and Aquatic Research Center, Lafayette, Louisiana, United States of America; 5 The Nature Conservancy in Florida, Maitland, Florida, United States of America; 6 U.S. Geological Survey, Louisiana Cooperative Fish and Wildlife Research Unit, School of Renewable Natural Resources, Louisiana State University Agricultural Center, Baton Rouge, Louisiana, United States of America; 7 U.S. Geological Survey, Wetland and Aquatic Research Center, Lafayette, Louisiana, United States of America; 8 Gulf Restoration, U.S. Fish and Wildlife Service, Department of the Interior, Lafayette, Louisiana, United States of America; 9 Mississippi State University, Coastal Research and Extension Center, Grand Bay National Estuarine Research Reserve, Moss Point, Mississippi, United States of America; University of Sydney, AUSTRALIA

## Abstract

Vulnerability assessments combine quantitative and qualitative evaluations of the exposure, sensitivity, and adaptive capacity of species or natural communities to current and future threats. When combined with the economic, ecological or evolutionary value of the species, vulnerability assessments quantify the relative risk to regional species and natural communities and can enable informed prioritization of conservation efforts. Vulnerability assessments are common practice in conservation biology, including the potential impacts of future climate scenarios. However, geographic variation in scenarios and vulnerabilities is rarely quantified. This gap is particularly limiting for informing ecosystem management given that conservation practices typically vary by sociopolitical boundaries rather than by ecological boundaries. To support prioritization of conservation actions across a range of spatial scales, we conducted the Gulf Coast Vulnerability Assessment (GCVA) for four natural communities and eleven focal species around the Gulf of Mexico based on current and future threats from climate change and land-use practices out to 2060. We used the Standardized Index of Vulnerability and Value (SIVVA) tool to assess both natural community and species vulnerabilities. We observed greater variation across ecologically delineated subregions within the Gulf Coast of the U.S. than across climate scenarios. This novel finding suggests that future vulnerability assessments incorporate regional variation and that conservation prioritization may vary across ecological subregions. Across subregions and climate scenarios the most prominent threats were legacy effects, primarily from habitat loss and degradation, that compromised the adaptive capacity of species and natural communities. The second most important threats were future threats from sea-level rise. Our results suggest that the substantial threats species and natural communities face from climate change and sea-level rise would be within their adaptive capacity were it not for historic habitat loss, fragmentation, and degradation.

## 1.0 Introduction

Anthropogenic climate change poses a major threat to many species and natural communities [1]. Due to threats from climate change and other factors, conservation agencies and organizations are often tasked with assessing threats to species and natural communities and prioritizing conservation actions. Vulnerability assessments are tools that can be used in this process [2]. Often, these assessments are executed at small spatial and short temporal scales that reflect the constraints and priorities of the executing agency more than they reflect biology and extirpation or extinction risk [3–5]. We conducted a multi-scale, multi-species and multi-habitat vulnerability assessment within a global biodiversity hotspot (northern Gulf of Mexico, USA)[6] to assess and help prioritize conservation efforts and to investigate the degree to which spatio-temporal scale impacts assessment and prioritization. Previous assessments in the Gulf region have focused on the state or regional-level [7] and none explicitly combined climate change, sea-level rise, and traditional land-use threats into a single vulnerability assessment.

Numerous methods exist for assessing the vulnerability of species and natural communities [3] and using that information in a prioritization process [8–11]. Many of these methodologies assess vulnerability to a specific threat, such as the impact of land-use or climate change, or separately evaluate the costs and logistics of potential mitigation strategies. For these tools to be effective for prioritization, there must be a way for individual assessments to be holistically considered in the decision-making process. Tools such as the Standardized Index of Vulnerability and Value (SIVVA) [11, 12] are modular and have the capacity to include multiple metrics such as vulnerability and value into a single prioritization system. SIVVA provides an easy to use instrument that can be applied at the species or natural community level, providing the flexibility to use the same tool with multiple units of assessment. The SIVVA framework also quantifies the relative value that species might possess to aid in their prioritization. Values include elevated priority if, for example, a species is evolutionarily unique, economically important, locally endemic, or of ecological importance as a highly interactive species [13]. Previous applications of SIVVA include statewide assessments in Florida [11, 14] and Georgia [15].

We conducted a multi-scale, multi-species and multi-habitat vulnerability assessment of the northern Gulf of Mexico to enhance conservation planning and implementation while supporting the goals of regional stakeholders. The Gulf Coast Vulnerability Assessment (GCVA) used the SIVVA framework to assess the vulnerability of four natural communities and a set of eleven associated focal species to develop a prioritization framework for conservation efforts that examined multiple threats. This effort is the first to assess and compare vulnerabilities for the same species within and across multiple spatial scales and future scenarios, and one of the first systematic vulnerability assessments to cross multiple political (US state) boundaries. We addressed three questions that have application within and beyond our study area: 1) Do vulnerabilities and priority rankings vary by region? 2) Which factors drive vulnerability and priority rankings in this region? Globally, land-use patterns are the primary driver of extinctions and extinction risk [16–19], but in coastal regions sea-level rise may play a larger role [11]. 3) Do conservation values and the way different types of information is weighted matter? Previous work has shown relatively modest impacts of alternative value systems as reflected by differential weighting of vulnerability and value criteria [11, 15].

## 2. Materials and methods

Experts provided assessments on natural communities and species using the SIVVA tool (section 2.1). We first outlined the study area of interest (section 2.2), defined the natural communities and species (section 2.3) of interest, and developed multiple maps and scenarios (section 2.4) to inform expert assessments. Statistical analyses relative to our hypotheses are given in section 2.5.

### 2.1.1 SIVVA criteria

Separate applications of the SIVVA tool were used to evaluate species and natural communities. For species, we used SIVVA as described in [11, 12]. A table of criteria used in the SIVVA for species analysis is available in S1 Table. For natural communities, we used a separate incarnation of the SIVVA tool designed especially for natural communities [14]. A description of the SIVVA for natural communities (SIVVA NATCOM) assessment tool can be found in S1 Table and elsewhere [14]. Both versions have the same scoring system within a Microsoft Excel spreadsheet. Experts are given specific guidelines for each criterion on how to provide a numerical score between 0 and 6. In this scoring system, a 0 means that not enough information is available; a score of 1 or 2 means positive impacts; a score of 3 means no impact; and a score of 4, 5, or 6 means increasingly negative impacts.

Criteria within each module of SIVVA are weighted and weights may be adjusted. A summary score was computed for each module by multiplying the weight of the criteria by the score from 1 to 6 and normalizing by the maximum total number of points, which creates a value on a scale from 0 to 1. An overall prioritization score was tallied by averaging across modules. Computing a mean score across all modules with equal weighting assumes that information on status, vulnerability, adaptive capacity, and conservation value should all be weighed equally in a prioritization scheme. A variety of value schemes exist in the field of conservation biology [20]; to reflect these differences we present four alternative weighting systems that differentially weight the score from each module (see Results). These alternative weighting schemes are one way of examining how variable priorities are under different value systems that might, for example, elevate the importance of conservation value over vulnerability, or vice versa.

In SIVVA NATCOM there are three modules: Ecosystem Status, Vulnerability, and Conservation Value. The Ecosystem Status module draws heavily from the IUCN framework [21, 22]. Ecosystem status includes three sets of criteria, of which the set with the highest score (the worst status) is counted and the others are ignored (S1 Table). The first set of criteria assesses the decline in area over the last fifty years, since 1750 (pre-Columbian era), and over any 50-year period including the present and future. The second set of criteria assesses the decline in ecosystem function over the same three timeframes. The third set of three criteria assesses the rarity of an ecosystem type with a focus on important differences between geographic extent, area of occupancy, and total area. These differences address the subtleties of how area is calculated; for example, several small, isolated habitat patches that form the same area as fewer large and continuous patches.

The second module is Vulnerability and includes nine criteria (S1 Table). These include quantitative estimates of area loss due to sea-level rise and land use change. Qualitative assessments include the impacts of fragmentation, alteration of disturbance regime, altered hydrology, inherent or imposed limits on range shifts, degradation of the abiotic environment, and other factors that would alter biotic processes and interactions.

The third module is Conservation Value and includes three criteria. These criteria assess the endemism of the community type, the number of highly interactive or rare species within that community, and the ecosystem services provided. The benefits of SIVVA NATCOM over existing assessments is that while it includes all of the major categories of existing tools, it also standardizes the score (a number between zero and one), provides a flexible framework for weighing different types of information differently, and is transparent in the way that different information is valued.

In SIVVA for Species there are four modules. The Vulnerability (Exposure + Sensitivity) module contains twelve criteria that address threats such as habitat loss to sea-level rise, erosion, and land use change, and species sensitivity to temperature, precipitation, and salinity changes. The Adaptive Capacity module contains six criteria that address intrinsic characteristics of the species that may allow it to cope with projected changes, such as species mobility, genetic diversity, and ability to colonize new areas. The Conservation Value module includes five criteria on endemism, evolutionary distinctiveness, ecological interactions, ecosystem services, and recovery potential. The Information Availability module includes five criteria on the data available for a species’ life history and documented responses to threats. The criteria are explained further in [11, 12].

### 2.1.2 SIVVA execution

The GCVA used expert opinion that was gathered through SIVVA. The vulnerability of each natural community and associated species was evaluated by subregion and climate scenario (see below), excluding those subregions where the species did not occur in significant numbers as reported by the assessors. Because vulnerability can vary with life-stage for many species, assessors were asked to consider the most vulnerable life-stage of the species for each criterion scored.

### 2.1.3 Expert engagement

The SIVVA tool requires input from species and natural community experts. For a given species or natural community in a particular subregion, an assessment ‘set’ was completed consisting of separate responses for each of three climate scenarios, which are described in more detail below. Assessors were recruited by team leads into four Ecosystem and Species Expert Teams (ESET) that were organized around the mangrove, tidal emergent marsh, oyster reef, and barrier island ecosystems. Assessors included individuals responsible for managing ecosystems or species and topical experts who had published extensively on a particular ecosystem or species.

At least two independent assessments (each of which included all climate scenarios) were completed in each ecosystem and species for each of the six subregions with requests by email and phone calls until a minimum of two independent assessments was achieved.

### 2.2 Study area

**Fig 1:**
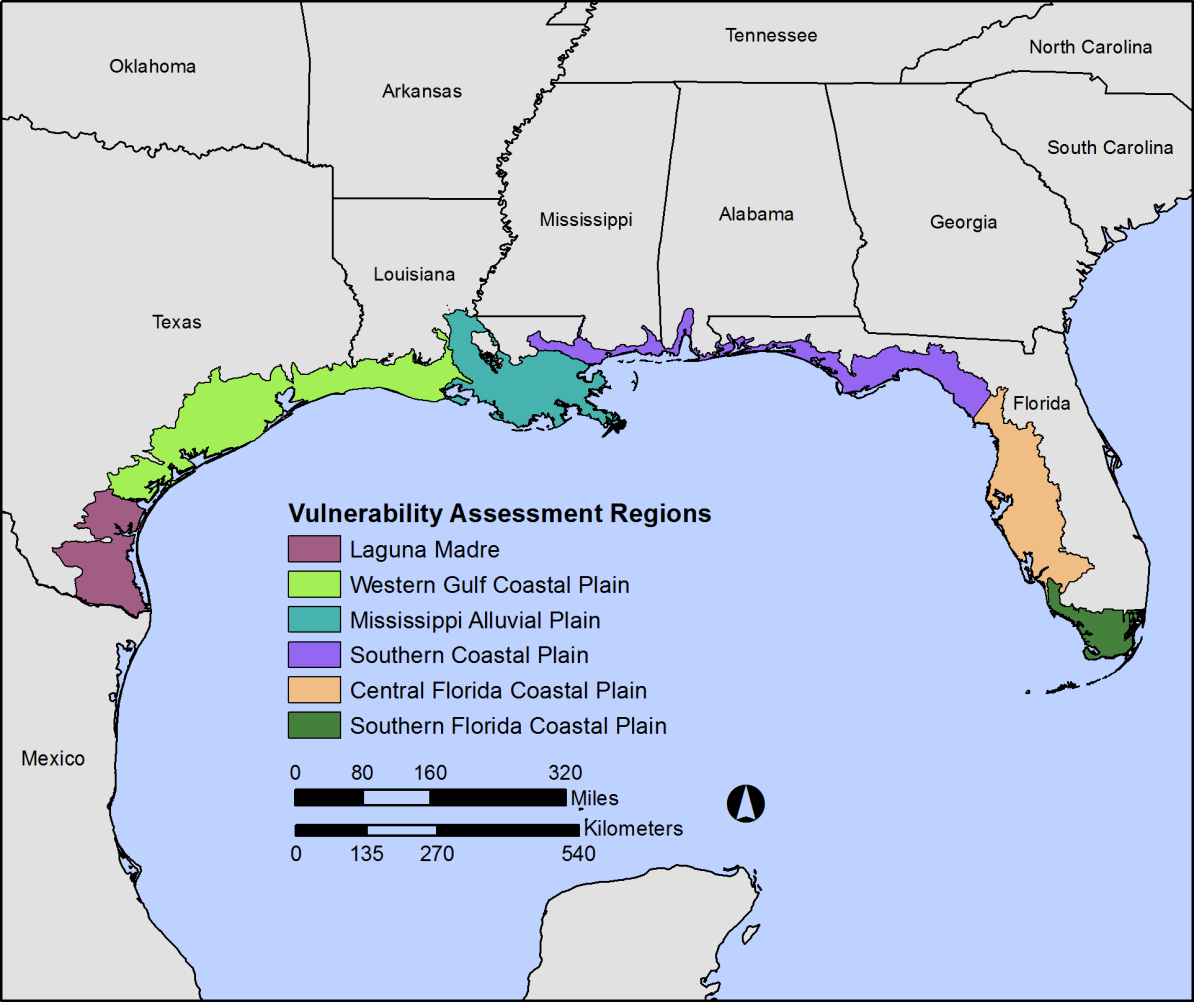
Six regions analyzed in Gulf Coast Vulnerability assessment.

The GCVA was focused on the northern Gulf of Mexico and spanned multiple state jurisdictions (Fig 1). Subregions were defined using Environmental Protection Agency (EPA) ecoregions [23–25] modified with the creation of the following subdivisions: a new Laguna Madre subregion was parsed out of the Western Gulf Coastal Plain from Corpus Christi, Texas, to the south due to a steep precipitation gradient occurring at that location; and a Central Florida Coastal Plain subregion was parsed out of the Southern Coastal Plain ecoregion from the Suwannee River in Florida to the north due to a shift in the dominance of mangroves. We limited the ecoregions to the National Oceanic and Atmospheric Administration (NOAA) Coastal Drainage Areas (CDAs) and Estuarine Drainage Areas (EDAs) hydrologic boundaries to include only those areas within the terrestrial ecoregions that are connected to Gulf Coast waters or estuaries.

The GCVA similarly used marine ecoregions defined for the Gulf of Mexico continental shelf, which extends from the coastline to the continental slope (steep transition to the deep sea) [26]. The GCVA clipped the marine ecoregions to the 30-meter isobath to those areas close to shore.

### 2.3 Natural communities and focal species

The Coastal and Marine Ecological Classification Standard (CMECS) provides a national framework for defining coastal and marine ecosystems based on their physical, biological, and chemical data [27]. We selected four natural communities for focus: mangroves, tidal emergent marsh, oyster reefs, and barrier islands, and 11 species dependent on at least one of these communities (Table 1). These natural communities and their focal species (Table 1) were chosen after several workshops with local management agencies because of their social, economic, ecological, and evolutionary importance, as well as availability of data and models to inform the assessment. Assessing both natural communities and associated focal species allows the GCVA to make recommendations that are both species specific and holistic to multispecies assemblages in the form of natural communities. Each natural community and its focal species are described in detail in S1 File.

**Table 1:** List of natural communities and their focal species. The number of independent assessments for each community and focal species are given in parentheses.

Natural Community	Focal Species
mangrove (33)	
	Bird: roseate spoonbill (*Platalea ajaja*) (21)
tidal emergent marsh (25)	
	Invert: blue crab (*Callinectes sapidus*) (27)
	Bird: clapper rail (*Rallus crepitans*) (25)
	Bird: mottled duck (*Anas fulvigula*) (21)
	Fish: spotted seatrout (*Cynoscion nebulosus*) (18)
oyster reefs (28)	
	Invert: eastern oyster (*Crassostrea virginica*) (37)
	Bird: American oystercatcher (*Haematopus palliatus*) (32)
	Fish: red drum (*Sciaenops ocellatus*) (21)
barrier islands (31)	
	Bird: black skimmer (*Rynchops niger*) (31)
	Reptile: Kemp’s ridley sea turtle (*Lepidochelys kempii*) (14)
	Bird: Wilson’s plover (*Charadrius wilsonia*) (23)

### 2.4.1 Timeframe

The year 2060 was chosen to assess future conditions because it coincides with other projects along the Gulf Coast such as the Southeast Conservation Adaptation Strategy [28], Florida Statewide Climate Scenarios [29], and the State of Louisiana’s Coastal Master Plan for 2012. If projections for 2060 were not available for a given model, the closest time step available was used, which for sea-level rise scenarios was 2050.

### 2.4.2 Scenarios and background data

All individuals conducting assessments were provided the same spatially varying projections of climate, sea-level rise, species and ecosystem distributions, urbanization projections, and conservation lands maps pertaining to the subregions that are outlined below. Maps containing data layers showing species and ecosystem distributions, sea-level rise projections, urbanization projections, and conservation lands were created on the Conservation Planning Atlas v. 2014 (http://gcplcc.databasin.org.). Information about each data source is provided below.

### 2.4.3. Present day species and natural community distribution data

Distribution maps of species and natural communities were generated for background information for SIVVA experts, using the best available sources. Barrier islands in the study area were delineated by the Ocean Conservancy in 2013 using an imagery service database of natural color imagery from years 2001 to 2011 [30]. Mangrove distribution was based on a published habitat model for current extent [31]. The data presented for tidal emergent marsh were the Estuarine Emergent Wetland land cover class from NOAA’s Coastal Change Analysis Program (C-CAP) 2010 Regional Land Cover Data for the Gulf of Mexico states. Consistent with our previous definition, Estuarine Emergent Wetlands are characterized by erect, rooted, herbaceous hydrophytes (excluding mosses and lichens) that are present for most of the growing season in most years. All water regimes are included except those that are subtidal and irregularly exposed [32]. This data set did not include freshwater tidal marsh distributions and a suitable source of recent data with adequate spatial resolution and excluding non-tidal marshes could not be found. Therefore, assessors were also asked to rely on their own knowledge of freshwater tidal marsh distribution to complement the provided maps of Estuarine Emergent Wetlands as they completed the assessment. Locations of oyster communities in the Gulf of Mexico were obtained from the 2011 Oyster dataset provided by NOAA's National Coastal Data Development Center [33]. These data represent side scan sonar-based data for oyster reefs within Gulf of Mexico estuaries. In some estuaries, particularly those in Louisiana, this dataset may underestimate living oyster reefs due to a lack of data for estuaries outside of publicly managed oyster seed grounds.

The bird species distribution maps for mottled duck, clapper rail, American oystercatcher, roseate spoonbill, black skimmer, and Wilson’s plover were obtained from BirdLife International and NatureServe. The distribution map for the Kemp’s ridley sea turtle was obtained from The State of the World’s Sea Turtles [34]. The Kemp’s ridley nest site summary for 2009 was obtained from the Bi-national recovery plan for the Kemp’s ridley sea turtle, second revision [35]. Nesting locations for Florida for 2009–2013 were acquired from the Statewide Nesting Beach Survey program coordinator of the Florida Fish and Wildlife Conservation Commission’s Fish and Wildlife Research Institute. Distribution maps of fish species available for the Gulf of Mexico did not provide adequate spatial resolution in the subregions of interest. Therefore, species experts were asked to rely on their own knowledge of red drum and spotted seatrout distribution for the assessment.

### 2.4.4. Spatial data on conservation lands

Dynamic web maps containing data layers showing species and natural community distributions, habitat projections, urbanization projections, and conservation lands were created on the Conservation Planning Atlas v. 2014 (http://gcplcc.databasin.org.). These data were shared with the species and habitat experts to assist in their evaluations. Conservation areas were identified using Version 2 of the Conservation Biology Institute Protected Areas Database (PAD-US, CBI Edition) available at http://consbio.org/products/projects/pad-us-cbi-edition.

### 2.4.5. Projected climate scenarios

For each subregion, we developed climate projections of seasonal averages for precipitation and air temperature, and developed three scenarios based on project CO_2_ emissions and sea-level rise taken from Intergovernmental Panel on Climate Change (IPCC) scenarios outlined below. Air temperature and precipitation projections were based on three combinations using the A2 and B1 climate scenarios produced by the Intergovernmental Panel on Climate Change [36]. These scenarios bracket uncertainty related to economic growth and global versus local sustainability efforts.

Sea surface temperature (SST) and surface ocean salinity (SOS) came from Representative Concentration Pathways (RCPs) scenarios 2.6 and 8.5 in the IPCC Fifth Assessment Report (AR5) [37]. These data were not available for the A2 and B1 emission scenarios, but RCP 8.5 and RCP 2.6 are emissions-conservative estimates relative to the A2 and B1 emission scenarios, respectively.

Sea-level rise rates were taken from within the range of possible future scenarios described in the Global Sea Level Rise Scenarios for the United States National Climate Assessment [38]. Sea-level rise amounts reported as 1.0 m and 2.0 m by 2100 were adjusted to 0.41 m and 0.82 m for the year 2050, which was as close as possible to the SECAS 2060 timeframe. Assessors were asked to evaluate species and natural community vulnerability under three different scenarios: 1) a low CO_2_ emissions (B1 and RCP 2.6) and low (0.41 m) sea-level rise; 2) a low CO_2_ emissions (B1 and RCP 2.6) and high (0.82 m) sea-level rise; and 3) a high CO_2_ emissions (A2 and RCP 8.5) and high (0.82 m) sea-level rise. For each subregion, we provided assessors with climate projections of seasonal averages for precipitation and air temperature (S1 Fig). Downscaled precipitation and air temperature projections from climate models used in the IPCC Fourth Assessment were obtained from [39]. Climate summaries for SST and SOS were provided for the entire seaward boundary as identified in Fig 1. For all climate parameters, climate projections for 2050–2069 were averaged and compared to the base period 1980–1999 [40].

### 2.4.6. Projected urban growth for the Gulf Coast

We used projections of urban growth for 2060 from a high-resolution regional probabilistic projection for the Southeast U.S. that was modified and implemented by the Biodiversity and Spatial Information Center at North Carolina State University using the Slope, Land cover, Exclusion, Urbanization, Transpiration, and Hillshade (SLEUTH) urban growth model [41, 42]. The projections focus on a current policy scenario and recent patterns of urban growth in the Southeast, typified by rapidly expanding low-density residential and commercial development. The model combines remotely sensed and transportation network data to capture observed patterns of suburban-exurban growth; the data presented for the SIVVA assessors were areas in the northern Gulf of Mexico with a 50% or greater probability of being urban in 2060.

### 2.4.7. Projected changes in habitat distribution due to sea-level rise

Projections for habitat distributions came from an application of the Sea Level Affecting Marshes Model (SLAMM) [43, 44]. The GCVA used results of three combinations of time step and eustatic sea-level rise scenario model outputs: initial condition, 0.41 m, and 0.82 m sea-level rise for 2050, which corresponds with the climate change scenarios above. Each SLAMM dataset was comprised of the 23 individual SLAMM (version 6) runs from across the Gulf Coast available at time of the assessment. Land cover types pertinent to this assessment were extracted and reclassified from the original 23 initial types to the 4 related to this project.

### 2.5. Statistics and analyses

Natural Community and focal species vulnerability values were reported using a baseline equal weghting scheme that factors criteria in each module equally. The influence of individual assessors on the resulting scores was evaluated by comparing their mean scores to the mean valuation across all assessors and by ensuring that variation across assessors for the same species or natural community was less than the mean variation across all assessments of species or natural communities, respectively.

To address our first question we determined how much variation in SIVVA scores was due to climate scenarios versus geographic subregions using a factorial analysis of variance (ANOVA). To address our second question we identified drivers of vulnerability rankings by listing the vulnerability criteria most highly ranked for each natural community and focal species. We addressed our third question about conservation values and the implications of weighing different types of information unequally by re-ranking natural communities and species under four distinct weighting schemes and assessing the impact on prioritization ranks.

Two types of uncertainty were accounted for: (1) scoring uncertainty, when an expert thinks more than one value is likely; and, (2) insufficient knowledge due to limited data available for the species. These two types of uncertainty are distinct: scoring uncertainty reflects uncertainty about the impacts of a vulnerability, whereas insufficient knowledge reflects a lack of information about a source of vulnerability. There may be limited direct life history data available for a given species, but the implications of sea-level rise for that species might be highly certain; for example a rare orchid may be poorly known to science but no orchids thrive in salt water inundation (high certainty but low information availability). Alternatively, a species may have high information availability, such as the well-studied Gulf Sturgeon, but low certainty about how this species will respond to sea-level rise. To account for scoring uncertainty, assessors could check a box next to the criterion to show they are not sure of the proper score, which is a value between 1 and 6. When this box is checked, in the final score computation, 0, +1, or -1 is added to the score that is marked as uncertain, and 1000 Monte Carlo simulations are run to recalculate the effect on the overall score. For example, if the user chooses a value of 3.5 for one criterion, the final score calculation measures the impact of 1000 simulations where that score is changed to 3.5 + 1 = 4.5, 3.5–1 = 2.5, or 3.5 + 0 = 3.5 to assess the variation on the summary score. If that criterion is heavily weighted, this uncertainty will impact the final summary score much more than if it is not heavily weighted, such that uncertainty about a fairly trivial criterion is not equivalent to uncertainty about a critical criterion. Insufficient knowledge is accounted for by reporting the proportion of criteria scored and by comparing the summary score to the proportion calculated as the total points divided by the maximum possible points available if all criteria had been scored.

Our assessments included both quantitative and qualitative scores. We elected to treat SIVVA scores as quantitative variables because they conformed to normality according to Shapiro-Wilks test. However, our results are qualitatively unchanged when SIVVA scores are treated as qualitative data and comparable non-parametric tests are executed.

## 3. Results

### 3.1. Assessor variability

Experts scored natural communities and species with high consistency, which we attributed to the use of standardized reference material and detailed scoring instructions [12]. All but 2 of the 27 assessors for natural communities fell within the 95% confidence interval of the mean scores (S2 File), which suggests relatively few assessors who scored consistently high or low regardless of the taxa they assessed. Neither of these two outlier assessors were the only assessor for a given community, such that their abnormally high or low scores were tempered by being averaged with multiple other assessors. All 48 assessors in the species assessment fell within the 95% confidence interval for all assessors over all regions. Our overall results are qualitatively unchanged when data from these two assessors are removed. More importantly, the variation among assessors for the same taxon or community was without exception less than the two standard deviations above or below the mean variance among all assessors.

### 3.2.1 SIVVA results for natural communities

**Fig 2:**
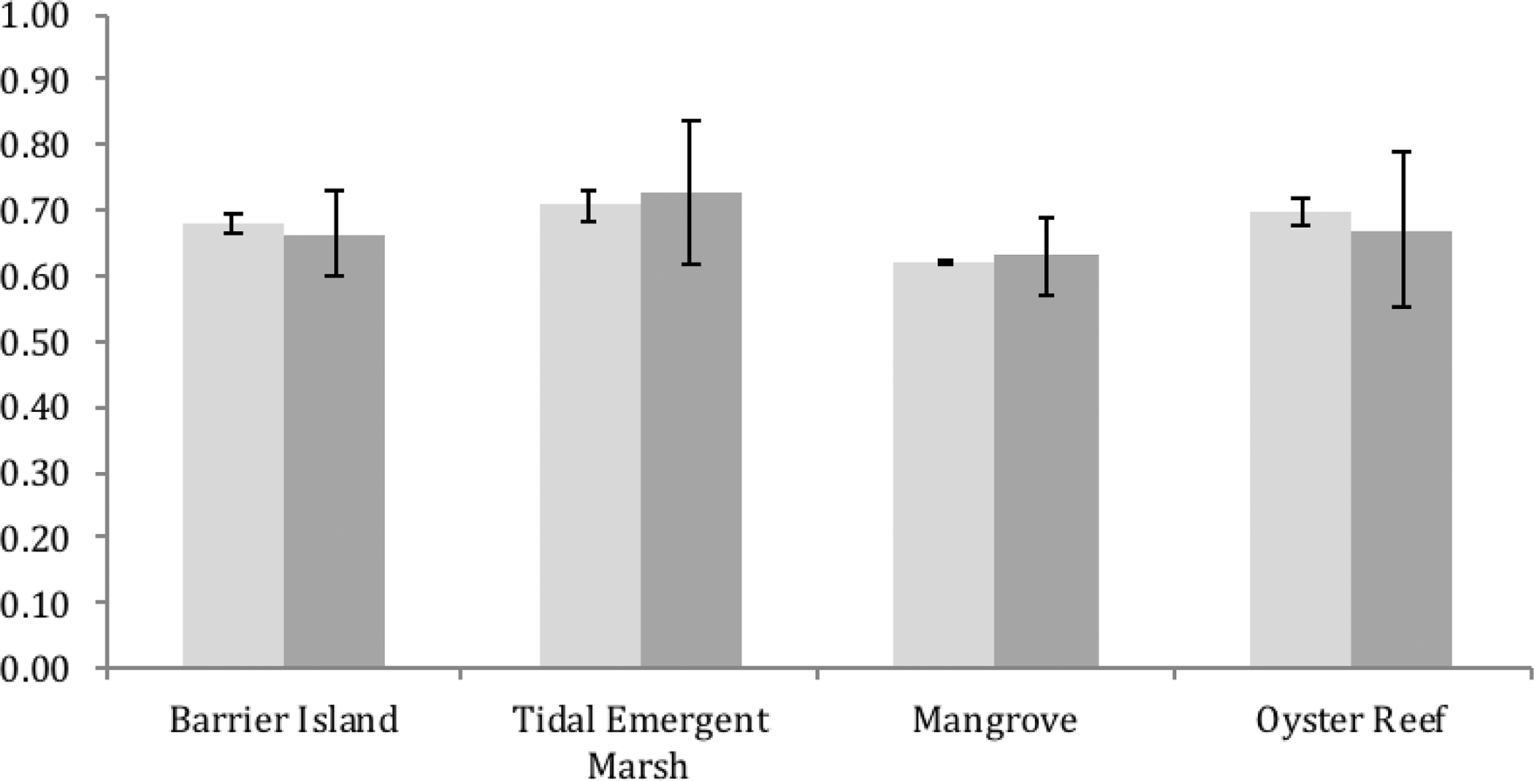
Ecosystem Status and Vulnerability SIVVA for natural community scores: Light grey bars are averaged across subregions with whiskers depicting variation across three climate scenarios; darker grey bars are averaged across climate scenarios with whiskers depicting variation across subregions. Note comparable scores, but greater variation across regions than climate scenarios.

**Fig 3:**
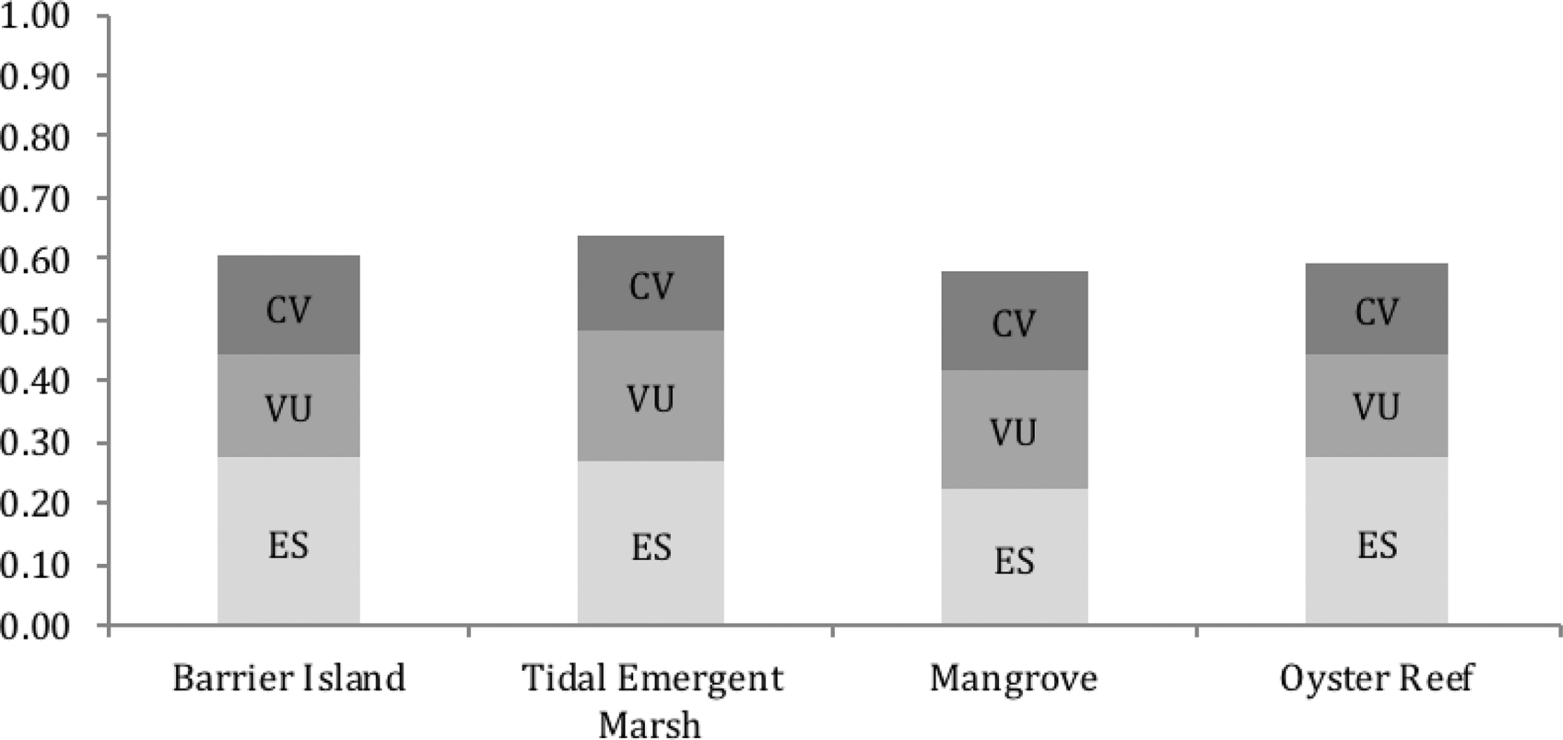
Overall SIVVA for natural communities scores averaged equally across modules, climate scenarios, and regions. Each module’s contribution to the overall score is given for Ecosystem Status (ES), Vulnerability (VU), and Conservation Value (CV).

The four natural communities showed more variation in Vulnerability and Ecosystem Status scores across regions than across climate scenarios (Fig 2) (statistics in section 3.3.1). Ecosystem Status scores were significantly higher than Vulnerability scores according to a paired t-test (31 df, P < 0.0001) (Fig 3, Table 2). Within Ecosystem Status, the three criteria that were consistently highest were (from highest to lowest): 1) decline in area since Industrial Revolution (1750); 2) decline in ecosystem function since Industrial Revolution; and, 3) the total extent of the natural community (a metric of rarity). The Vulnerability module included projected impacts from future land-use and climate change, but the Ecosystem Status module scores primarily reflect historical and contemporary human land-use. The tendency for higher Ecosystem Status scores relative to Vulnerability scores suggest that prospects for natural communities in this area are more determined by historical factors, typically human land-use patterns, than by vulnerabilities to future threats from climate change (or future land-use patterns). Detailed results for each natural community and focal species are given in S3 File. For the Vulnerability module, four of the nine criteria consistently ranked high, including losses of area due to sea-level rise, fragmentation of habitat patches, altered hydrology, and degradation of abiotic habitat features. For the Conservation Value module, the criterion related to ecosystem services clearly weighed most heavily, although our strategy for choosing which natural communities to assess makes this an expected outcome.

**Table 2:** Results of SIVVA assessments for natural communities for all three climate scenarios and across all six regions. Scores are presented for all three modules: Ecosystem Status (ES), Vulnerability (VU), and Conservation Value (CV).

Natural Community	ES	VU	CV
**Barrier Islands**			
Climate Scenario 1	0.86	0.50	0.49
Climate Scenario 2	0.84	0.48	0.47
Climate Scenario 3	0.84	0.54	0.47
Laguna Madre	0.72	0.39	0.61
Western Gulf Coastal Plain	0.89	0.48	0.72
Mississippi Alluvial Plain	0.96	0.57	0.52
Southern Coastal Plain	0.76	0.47	0.33
Central Florida Coastal Plain	0.86	0.49	0.44
Southern Florida Coastal Plain			
**Tidal Emergent Marsh**			
Climate Scenario 1	0.78	0.59	0.64
Climate Scenario 2	0.78	0.63	0.67
Climate Scenario 3	0.79	0.67	0.70
Laguna Madre	0.69	0.53	0.47
Western Gulf Coastal Plain	0.72	0.58	0.50
Mississippi Alluvial Plain	0.86	0.65	0.56
Southern Coastal Plain	0.75	0.54	0.32
Central Florida Coastal Plain	0.78	0.48	0.50
Southern Florida Coastal Plain	1.00	0.80	0.67
**Mangroves**			
Climate Scenario 1	0.66	0.58	0.47
Climate Scenario 2	0.66	0.58	0.47
Climate Scenario 3	0.66	0.58	0.47
Laguna Madre	0.67	0.72	0.50
Western Gulf Coastal Plain	0.64	0.54	0.42
Mississippi Alluvial Plain	0.63	0.54	0.42
Southern Coastal Plain	0.64	0.48	0.38
Central Florida Coastal Plain	0.72	0.69	0.42
Southern Florida Coastal Plain	0.69	0.60	0.59
**Oyster Reef**			
Climate Scenario 1	0.83	0.52	0.44
Climate Scenario 2	0.86	0.55	0.44
Climate Scenario 3	0.86	0.56	0.44
Laguna Madre	0.78	0.70	0.44
Western Gulf Coastal Plain	0.87	0.59	0.44
Mississippi Alluvial Plain	0.92	0.64	0.44
Southern Coastal Plain	0.78	0.44	0.43
Central Florida Coastal Plain	0.89	0.31	0.44
Southern Florida Coastal Plain	0.67	0.26	0.44

### 3.2.2. SIVVA results for focal species

The species results generally reflected slightly higher scores for Vulnerability and Information Availability, and lower scores for Adaptive Capacity and Conservation Value (Table 3). Within the Vulnerability module the threats from sea-level rise were by far the highest scored forms of vulnerability. The criterion within Adaptive Capacity that was “the ability of species to shift in response to threats” was consistently the most influential criterion on the scores for the module. Similar to the assessments of natural communities, ecosystem services were the most influential component of the Conservation Value module. Lastly, the criterion that consistently ranked the highest in the Information Availability module was the availability of published literature on basic life history. In this module, high scores translate into high information availability and thus an elevated priority. This result was not surprising given that the high profile of the species we assessed typically meant that these species were also well-studied.

**Table 3:** Results of SIVVA assessments for species for all three climate scenarios (SC1-SC3) and across all six regions: Laguna Madre (LM), Western Gulf Coastal Plain (WGCP), Mississippi Alluvial Plain (MAP), Southern Coastal Plain (SCP), Central Florida Coastal Plain (CFCP), and Southern Florida Coastal Plain (SFCP). Scores are presented for all three modules: Ecosystem Status (ES), Vulnerability (VU), and Conservation Value (CV).

	V	AC	CV	IA
eastern oyster (*Crassostrea virginica*)				
SC1	0.59	0.44	0.53	0.72
SC2	0.62	0.46	0.52	0.73
SC3	0.67	0.47	0.52	0.73
LM	0.55	0.42	0.57	0.63
MAP	0.53	0.40	0.57	0.50
WGCP	0.59	0.46	0.55	0.69
SCP	0.64	0.45	0.52	0.80
CFCP	0.66	0.43	0.50	0.78
SFCP	0.49	0.41	0.50	0.78
red drum (*Sciaenops ocellatus*)				
SC1	0.52	0.31	0.49	0.75
SC2	0.60	0.31	0.43	0.75
SC3	0.63	0.33	0.43	0.75
LM	0.52	0.21	0.45	0.89
MAP	0.58	0.46	0.53	0.78
WGCP	0.55	0.33	0.49	0.83
SFCP	0.46	0.21	0.47	0.67
CFCP	0.52	0.21	0.47	0.67
SCP	0.46	0.38	0.53	0.58
mottled duck (*Anas fulvigula*)				
SC1	0.58	0.38	0.46	0.51
SC2	0.58	0.38	0.37	0.51
SC3	0.63	0.40	0.37	0.51
LM	0.49	0.45	0.44	0.54
MAP	0.57	0.38	0.43	0.53
WGCP	0.56	0.45	0.44	0.54
SFCP	0.58	0.29	0.50	0.42
CFCP	0.60	0.29	0.50	0.42
SCP	0.69	0.43	0.50	0.61
spotted seatrout (*Cynoscion nebulosus*)				
SC1	0.47	0.39	0.44	0.62
SC2	0.48	0.39	0.44	0.62
SC3	0.50	0.39	0.44	0.62
LM	0.42	0.31	0.40	0.50
MAP	0.59	0.41	0.45	0.56
WGCP	0.59	0.41	0.45	0.56
SFCP	0.35	0.40	0.43	0.75
CFCP	0.40	0.40	0.43	0.75
SCP	0.46	0.42	0.50	0.58
American oystercatcher (*Haematopus palliatus*)				
SC1	0.64	0.56	0.34	0.52
SC2	0.66	0.56	0.32	0.53
SC3	0.70	0.57	0.32	0.53
LM	0.48	0.55	0.32	0.50
MAP	0.60	0.43	0.31	0.59
WGCP	0.54	0.57	0.33	0.51
SFCP	0.84	0.75	0.35	0.58
CFCP	0.79	0.65	0.35	0.53
SCP	0.72	0.53	0.36	0.47
clapper rail (*Rallus crepitans*)				
SC1	0.59	0.45	0.34	0.57
SC2	0.58	0.45	0.36	0.55
SC3	0.61	0.45	0.36	0.55
LM	0.67	0.54	0.35	0.63
MAP	0.66	0.42	0.32	0.50
WGCP	0.57	0.54	0.35	0.63
SFCP	0.72	0.56	0.47	0.69
CFCP	0.51	0.38	0.35	0.54
SCP	0.54	0.43	0.32	0.56
blue crab (*Callinectes sapidus*)				
SC1	0.40	0.21	0.46	0.56
SC2	0.41	0.21	0.46	0.56
SC3	0.39	0.21	0.46	0.56
LM	0.41	0.20	0.48	0.47
MAP	0.39	0.27	0.40	0.73
WGCP	0.40	0.20	0.48	0.47
SFCP	0.40	0.17	0.50	0.58
CFCP	0.45	0.17	0.50	0.58
SCP	0.32	0.19	0.43	0.50
roseate spoonbill (*Platalea ajaja*)				
SC1	0.63	0.53	0.44	0.52
SC2	0.67	0.56	0.47	0.50
SC3	0.71	0.57	0.47	0.50
LM	0.49	0.54	0.42	0.60
MAP	0.64	0.36	0.40	0.38
WGCP	0.58	0.45	0.41	0.49
SFCP	0.56	0.47	0.46	0.55
CFCP	0.76	0.60	0.46	0.57
SCP	0.72	0.77	0.43	0.53
black skimmer (*Rynchops niger*)				
SC1	0.68	0.48	0.43	0.46
SC2	0.70	0.47	0.40	0.45
SC3	0.73	0.48	0.40	0.45
LM	0.60	0.58	0.40	0.50
MAP	0.74	0.38	0.43	0.45
WGCP	0.67	0.44	0.43	0.48
SFCP	0.71	0.56	0.45	0.44
CFCP	0.68	0.46	0.43	0.42
SCP	0.66	0.60	0.43	0.42
Wilson’s plover (*Charadrius wilsonia*)				
SC1	0.66	0.60	0.37	0.46
SC2	0.71	0.54	0.37	0.45
SC3	0.74	0.56	0.36	0.46
LM	0.55	0.61	0.37	0.45
MAP	0.71	0.45	0.38	0.47
WGCP	0.64	0.51	0.38	0.48
SFCP	0.64	0.92	0.32	0.47
CFCP	0.64	0.92	0.32	0.47
SCP	0.85	0.55	0.40	0.39
Kemp’s ridley sea turtle (*Lepidochelys kempii*)				
SC1	0.62	0.76	0.66	0.54
SC2	0.70	0.77	0.61	0.54
SC3	0.70	0.75	0.57	0.56
LM	0.50	0.71	0.70	0.64
MAP				
WGCP	0.71	0.85	0.58	0.53
SFCP				
CFCP				
SCP	0.70	0.76	0.66	0.45

**Fig 4:**
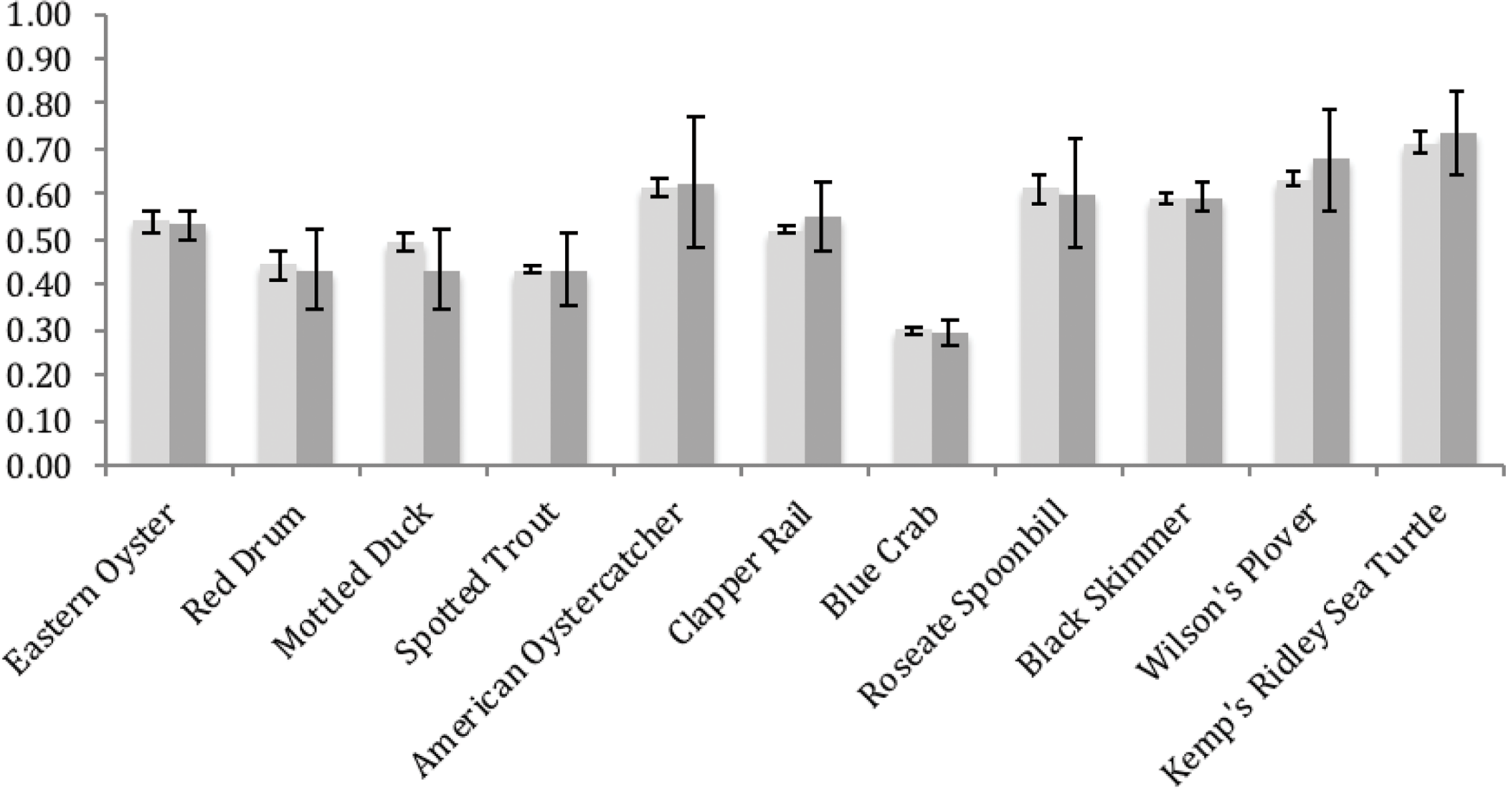
Vulnerability and adaptive capacity SIVVA for species scores: Light grey bars are averaged across subregions with whiskers depicting variation across three climate scenarios; darker grey bars are averaged across climate scenarios with whiskers depicting variation across subregions. Note comparable scores, but greater variation across regions than climate scenarios.

**Fig 5:**
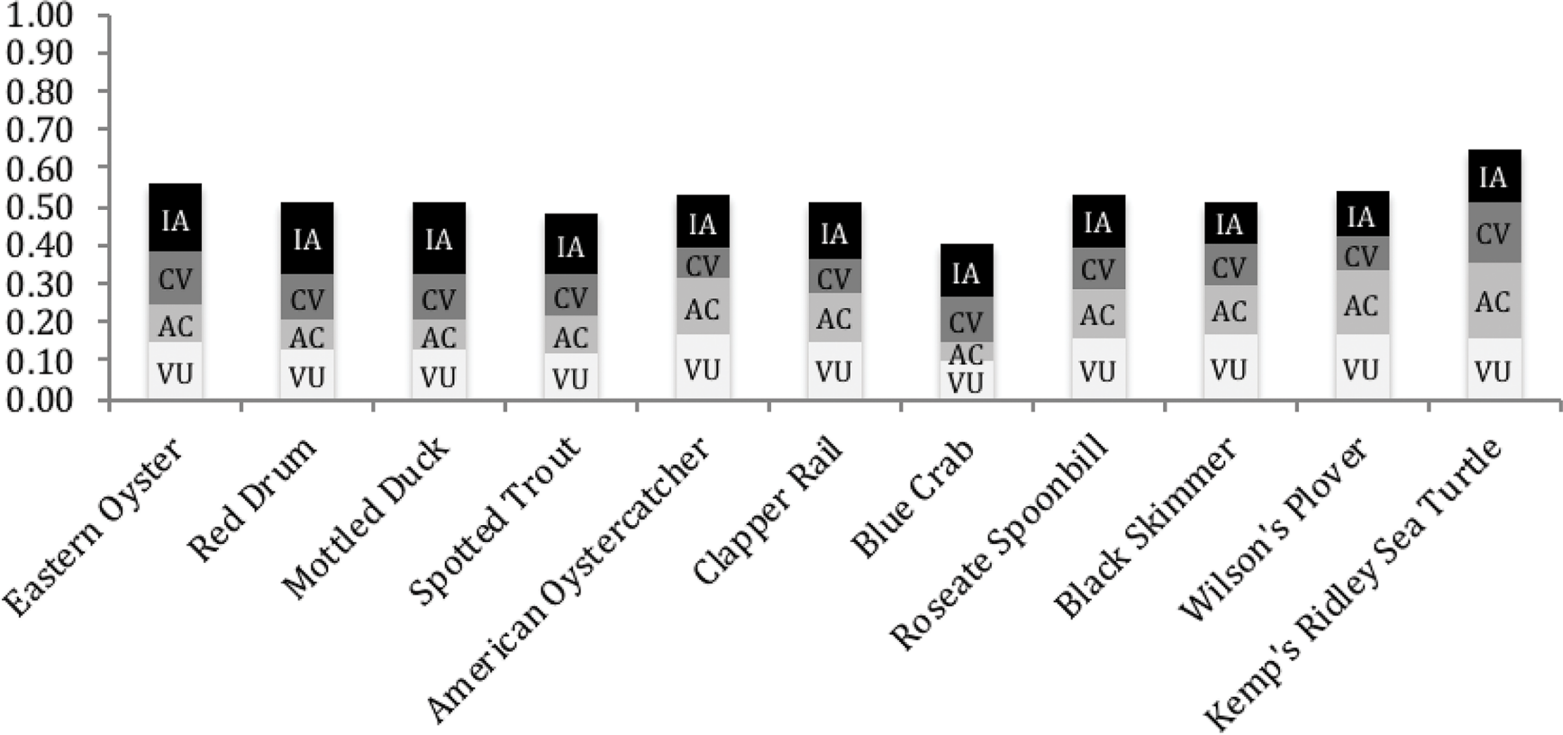
Overall SIVVA for species scores averaged equally across modules, climate scenarios, and regions. Each module’s contribution to the overall score is given for Vulnerability (VU), Adaptive Capacity (AC), Conservation Value (CV), and Information Availability (IA).

As with natural communities, species vulnerabilities and adaptive capacity showed little spatial variation by subregion, but high variability across climate scenarios (Fig 4; see section 3.3.2). Vulnerability scores tended to be higher than Adaptive Capacity or Conservation Value scores (Fig 5, Table 3). This suggests that species tend to have reasonably high adaptive capacity to some threats, but that the combined impact of land-use, climate change, and sea-level rise might exceed that capacity.

### 3.3.1. Variation by subregion for natural communities

The factorial ANOVAs empirically demonstrate greater variation by subregion than by climate area (Fig 2), and that a non-significant portion of variance in Ecosystem Status results from climate scenarios (P = 0.11, 0.01% of variation), compared to a highly significant result for subregions (P < 0.0001, 7.16% of variation). Climate scenario and subregion variability contributed significant but modest variation to Vulnerability (climate scenario: P < 0.0001, 0.51% of variation; subregion: P < 0.0001, 0.83% of variation). Tidal emergent marshes and oyster reefs showed the greatest variation across regions, potentially due to spatially varying relative sea-level rise and associated threats to water quality [45]. The southern Florida coastal plain and Mississippi alluvial plain were areas of exceptionally poor ecosystem status and high vulnerability for both oyster reefs and tidal emergent marshes, potentially due to extensive coastal development and low elevation profiles [46, 47].

### 3.3.2. Variation by subregion for species

Similar to natural communities, scores within and averaged across modules varied substantially more by region than by climate scenario (Fig 4). Vulnerability and Adaptive Capacity varied most, while there was little variation in Conservation Value or Information Availability across regions (Table 3). There were four species with noticeably more variation in Vulnerability and Adaptive Capacity across regions than all others: American oystercatcher, roseate spoonbill, Wilson’s plover, and Kemp’s ridley sea turtle. These four species also had among the highest overall SIVVA scores. All four showed high inter-region variation relative to other taxa. These results suggest that among the most vulnerable species, spatial heterogeneity in that vulnerability may be high and necessitate region-specific interventions. Similar to results for natural communities, factorial ANOVA revealed significant results with modest effect sizes for subregion and climate scenario for both Vulnerability (subregion: P < 0.0001, 3.5% of variation; climate scenario: P < 0.0001, 0.82% of variation) and Adaptive Capacity (subregion: P < 0.0001, 0.22% of variation; climate scenario: P < 0.0001, 0.26% of variation).

### 3.4. Relative contributions of different types of information to prioritization and the impacts of value systems

When each module in SIVVA NATCOM was weighted equally, the priority rankings of the four natural communities based on overall risk and value are: Tidal emergent marsh, barrier island, oyster reef, and mangrove. Four alternative value schemes were considered: Status (50% ES, 25% VU, 25% CV), Vulnerability (25% ES, 50% VU, 25% CV), Vulnerability only (100% VU), and Ecosystem Status and Vulnerability only (50% ES, 50% VU). Tidal emergent marsh was the highest ranked community across all four value systems, but rankings for the other natural communities shifted depending on weighting (Table 4).

**Table 4:** Rankings of natural communities across value schemes. Value schemes differ in how different types of information are weighted and are depicted as percentages (which sum to 100%) in the order of Modules: Ecosystem Status, Vulnerability, and then Conservation Value.

	Value Schemes				
Natural Community	33/33/33	50/25/25	25/50/25	0/100/0	50/50/0
barrier island	2	2	3	4	3
tidal emergent marsh	1	1	1	1	1
mangrove	4	4	2	2	4
oyster reef	3	3	4	3	2

Species assessments showed greater variation in priority rankings depending on the value system, as reflected by different weighting of the four modules (Table 5). The 11 species changed ranks by as many as 8 places (e.g., from 1 out of 11 to 9 out of 11), with an average of 4 places across all five value systems. This result suggests that while there is some consistency in how natural communities rank regardless of which types of information is emphasized (Ecosystem Status, Vulnerability, and/or Conservation Value), prioritization may be more variable for species. This variability requires clarity on the part of the prioritizing agency with respect to what types of information are most important for allocating conservation efforts. It should be noted, however, that in relatively short lists of species or natural communities, these shifts are modest in impact. For example, a list of four natural communities can of necessity only show small variation in rank scores.

**Table 5:** Rankings of species across value schemes. Value schemes differ in how different types of information are weighted and are depicted as percentages (which sum to 100%) in the order of Modules: Vulnerability, Adaptive Capacity, Conservation Value, and then Adaptive Capacity.

	Value Schemes					
Species	25/25/25/25	45/25/20/10	20/20/50/10	15/15/35/35	100/0/0/0	Range in Rank
eastern oyster	2	6	2	2	7	5
red drum	7	8	6	3	8	5
mottled duck	7	8	6	3	8	5
spotted trout	10	10	10	6	10	4
American oystercatcher	5	3	8	8	3	5
clapper rail	9	7	9	7	6	3
blue crab	11	11	11	11	11	0
roseate spoonbill	4	5	3	5	5	2
black skimmer	6	4	4	9	1	8
Wilson's plover	3	2	5	10	2	8
Kemp's ridley sea turtle	1	1	1	1	4	3

## 4.0 Discussion

### 4.1 Spatial variability is greater than climate model variability

The GCVA using SIVVA provides detailed results on the regional and system-wide threats facing major natural communities and focal species (S3 File). This work demonstrates significant regional variation in vulnerability, with little to no effect of climate model choice on the resulting assessment (Figs 2 and 4). This finding is important because climate model projections vary dramatically, particularly at timescales of 50 to 100 years into the future [48]. One consequence of this variability is that researchers frequently bracket the uncertainty in their assessments of extinction risk using low, mid-range, and high change climate models [49]. The variability across these models can be dramatic, resulting in projected global biodiversity losses from 18% at the low end to up to 35% of species diversity [19]. Ours is among the first to compare variation due to climate model to regional variation within the same species or natural community. The degree to which climate scenario matters less than regional spatial variability warrants further research, particularly given that our timeline extended to 2060 and most climate projections do not deviate from each other dramatically until between 2050 and 2100. Future research should assess both the impacts of climate model on extinction risk, vulnerability, and prioritization, but also the degree to which regionally distinct populations react to threats. In the case of the GCVA, that regional variation in adaptive capacity greatly exceeded variation due to climate models.

Among the future threats that species and natural communities face, we find that sea-level rise is a major and primary contributor to vulnerability. However, the presence of pathways to migrate is as or more important than the direct impacts of sea-level rise, and our assessment reveals significant regional variation in the presence of pathways to migrate inland or upslope. Thus, while the impacts of sea-level rise vary regionally due to tidally adjusted elevations and slope profiles [50], this variation may be negligible relative to differences in the adaptive capacity of species and communities to shift their distributions [51, 52]. We found that regional variation in land-use impacts, which may squeeze species “between the devil and the deep blue sea” are as or more important than regional variation in sea-level rise [53].

### 4.2 Historical habitat loss and degradation affect vulnerability and value more than projected future threats from climate change (or anything else)

The current status of all four natural communities was ranked by assessors as having a greater influence on future viability than the vulnerability to projected threats (Fig 3). It is possible that the legacy impacts of land-use since the Industrial Revolution are difficult to assess with as much precision as those of future threats, but in our case assessor uncertainty was low relative to the differences between current ecosystem status and future vulnerability. While changes in natural community extent since the Industrial Revolution have been extensive in the southeastern US [54], much of the southeastern coastal plain flora and fauna remained relatively stable for millions of years prior to the Industrial Revolution [6]. This stability prior to human influence suggests an innate adaptive capacity in species and natural communities to deal with climate and sea-level rise oscillations. Projected changes in climate and sea-level rise out to 2060 or even 2100 are not outside of the variation experienced in this region over the last 100,000 years [55–58]. Natural communities would therefore potentially be able to respond to expected climate change variability in the next century, but that innate ability is compromised by human land-use and loss of ecosystem extent and health. While climate change poses a serious threat to species and natural communities, the primary drivers of extinction risk are still the legacy effects of two centuries of habitat loss and degradation [59, 60].

Our assessment included a criterion on potential indirect impacts due to deteriorated abiotic conditions or the loss of key biotic interactions; however, the lack of data made this criterion difficult to evaluate for many assessors. This is a key area for future research because many extinctions linked to climate change are often indirect [61] as a result of the complexity of ecological interactions in natural systems. This and other data gaps, for example the lack of detailed spatial mapping of mangroves throughout much of Louisiana and Texas, are also a valuable product of this research and can be used for targeted programs that help fill these data gaps.

### 4.3 Conclusions

We address three questions that are broadly applicable outside of our study area in the Gulf Coast. First, we show that vulnerability to future threats varied significantly more by region than by climate scenarios, suggesting that future vulnerability assessments may need to evaluate the relative importance of geographic scale. Moreover, the common approach of adopting global or state rankings from the IUCN or NatureServe [62] for state or regional conservation planning may not reflect local conditions. Secondly, we demonstrated that the most variable criteria were the impacts of sea-level rise and, importantly, the potential to migrate as a potential response to sea-level rise or other encroachments. Third, we identified substantial variation in prioritization ranks across value systems representative of the broader conservation community for different stakeholders. Previous results using these methods revealed consistent rankings across these same sets of value systems [11, 12]. In those cases, the interpretation was that the species that ranked highly due to one threat or criterion, also ranked highly due to other threats and criteria. Because the threats were more homogenous in these previous assessments, differential weighting of criteria did not have a large effect. Rankings likely varied across values systems in this study because each species faced fairly unique threats. It is also possible that the variation in rankings among our species and natural communities was influenced by how we chose those entities; a random or more systematic selection process might yield different patterns, at least for species. The implication of this work for the broader conservation community is that variation in how information is weighted may or may not significantly influence the prioritization scores, particularly in the Gulf Coast. In the future, researchers should empirically assess the impact of their value systems on their resulting conservation prioritizations.

The GCVA allows for unique interpretations to be made for conservation actions given the hierarchical nature of our assessment, from species to the natural communities that contain those (and other) species. We find that the threats that focal species face are typically the same threats faced by the broader natural communities in which those species reside. Thus, our findings support the use of carefully chosen indicator and umbrella species for vulnerability assessments [63, 64]. We also found it striking that results with respect to regional variation in vulnerability relative to variability due to climate models were consistent in both our species and natural communities assessments- this pattern was not unique to a handful of species. The most important outcomes of this multilevel assessment for conservation are the specific threats identified (S3 File), which can be used to help guide conservation actions and mitigate conflicting interventions [65–67].

## Supporting information

S1 Table SIVVA NATCOM and SIVVA for species criteria and modules. The SIVVA modules (in italics) and criteria for each module are listed with descriptions and directions associated with the criteria. Differentially shaded groupings in Ecosystem Status indicate that only the highest scored criteria in the subsection will be used in final SIVVA scoring.
(DOCX)

S1 File Descriptions of natural communities. (DOCX)

S1 Fig Climate summaries and seasonal averages. (DOCX)

S2 File Assessor variation. (DOCX)

S3 File Results of vulnerabilities for natural communities and focal species. (DOCX)
