# Supplementary material for: A multiscale natural community and species-level vulnerability assessment of the Gulf Coast, USA
Source: PLoS One. 2018 Jun 29;13(6):e0199844. doi: 10.1371/journal.pone.0199844 (PMC6025860; doi:10.1371/journal.pone.0199844)
Supplement: S1 Table — The SIVVA modules (in italics) and criteria for each module are listed with descriptions and directions associated with the criteria. Differentially shaded groupings in Ecosystem Status indicate that only the highest scored criteria in the subsection will be used in final SIVVA scoring. (DOCX) [file pone.0199844.s001.docx]

S1 Table. SIVVA NATCOM and SIVVA for Species Criteria and Modules. The SIVVA modules (in italics) and criteria for each module are listed with descriptions and directions associated with the criteria. Differentially shaded groupings in Ecosystem Status indicate that only the highest scored criteria in the subsection will be used in final SIVVA scoring.

| SIVVA NATCOM Criteria | Criteria Descriptions and Instructions |
| --- | --- |
| *Ecosystem Status: Three subcategories- decline in area, decline in quality or ecosystem function, and overall rarity* | |
| 1. Historical (last 50 years) decline in area | Over the last 50 years, what is the decline in landcover (total area) for this community due to conversion to urban area or agriculture, or outright destruction? Degradation will be assessed in the next group of criteria. |
| 2. Historical (since 1750) decline in area | Since approximately 1750 (Industrial Revolution), what is the decline in landcover (total area) for this community due to conversion to urban area or agriculture, or outright destruction? Degradation will be assessed in the next group of criteria. |
| 3. Observed or predicted decline in extent over any 50 year period including present and future | What is the decline in extent (total area) for this community over any 50 year period including present and future due to conversion to urban area or agriculture, or outright destruction? Degradation will be assessed in the next group of criteria. |
| 4. Historical (last 50 years) decline in ecosystem function | Over the last 50 years, what is the decline in ecosystem function for this community? Ecosystem function is here defined as the physical, chemical, and biological processes or attributes that contribute to the self-maintenance of an ecosystem. Examples include primary production, nutrient cycling, energy and matter transfer, etc. Here and elsewhere, "severity" refers to likelihood of collapse, where 100% severity is assured ecosystem collapse. |
| 5. Historical (since 1750) decline in ecosystem function | What is the decline in ecosystem function for this community over any 50 year period including present and future? Ecosystem function is here defined as the physical, chemical, and biological processes or attributes that contribute to the self-maintenance of an ecosystem. Examples include primary production, nutrient cycling, energy and matter transfer, etc. |
| 6. Observed or predicted decline in ecosystem function over any 50 year period including present and future | What is the decline in ecosystem function for this community over any 50 year period including present and future? Ecosystem function is here defined as the physical, chemical, and biological processes or attributes that contribute to the self-maintenance of an ecosystem. Examples include primary production, nutrient cycling, energy and matter transfer, etc. |
| 7. Geographic extent- total extent | Imagine or draw a minimum convex polygon around all current occurrences of this community type. Please choose from the following options to characterize this polygon as the geographic extent of the natural community: |
| 8. Geographic extent- area of occupancy | Use the total extent map of the community being assessed to determine how many of those counties this habitat is present in significant acreage? |
| 9. Geographic extent- total acreage | What is the percentage of the study area encompassed by this habitat type? |
| *Vulnerability* | |
| 10. Proportion of community area likely to be lost directly to SLR | What proportion of the community extent is likely to be lost direction to 0.5 or 1 m of SLR by the SLAMM model projections? If unable to provide an empirical estimate, please choose from the following options: |
| 11. Proportion of community area likely to be lost to urbanization or other land-use change | What proportion of the community extent is likely to be lost directly to LUC as projected by the included map? If unable to provide an empirical estimate, please choose from the following options: |
| 12. Vulnerability to current or expected (by 2050) extent to fragmentation | Please characterize the impacts of SLR and LUC on fragmenting the extent of this natural community, for example, increased road-building might fragment a habitat without substantially decreasing total area or extent. |
| 13. Vulnerability of community to altered disturbance regime (e.g., altered fire regime or weather pattern), including management alterations, for example a shift from heterogeneous lightning fire to homogeneous prescribed burn | Over the last 50 years this area receives approximately 50 inches of rain per year. By 2050 models show annual precipitation of approximately 52.5 inches with little change in wet-dry season variability. By 2100 models show annual precipitation of approximately 55 inches with little change in wet-dry season variability. What are the impacts of changes in the future climate, land-use or sea level to hydrology, and how will those impacts affect the natural community? |
| 14. Vulnerability of the community to altered hydrology (salinity, water table, hydroperiod, etc.) | Over the last 50 years this area receives approximately 50 inches of rain per year. By 2050 models show annual precipitation of approximately 52.5 inches with little change in wet-dry season variability. By 2100 models show annual precipitation of approximately 55 inches with little change in wet-dry season variability. What are the impacts of changes in the future climate, land-use or sea level to hydrology, and how will those impacts affect the natural community |
| 15. Vulnerability of community to invasive species (list of major invasives) | Use the list of "Priority 1 Invasive Species" provided in the instructions to score the existing or potential impact of invasive species on these natural communities. Impacts should be judged in terms of extent, function, and ecosystem services. |
| 16. Constraints on range shifts | Evaluate the possibility that this community can shift its spatial extent in response to described threats from SLR, CC, and LUC (or other threats). Potential barriers to range shifts include strict edaphic requirements that do not exist elsewhere, natural barriers (e.g., open ocean), or man-made barriers (e.g., major metropolitan areas). The rate of change can be critical in this evaluation, so consider the 2100 timeframe. |
| 17. Other factors that would degrade abiotic environment | In addition to the threats described above, are there other threats that would degrade the abiotic environment and reduce carrying capacity or niche diversity (e.g., salinization of soil or destruction of soil or seed beds by offroad vehicles)? |
| 18. Other factors that would alter biotic processes and interactions | In addition to the threats described above, are there other threats that would alter biotic processes and interactions and further reduce vital rates and mutualisms or interfere with community dynamics (e.g., loss of keystone species or key pollinators)? |
| *Conservation Value* | |
| 19. How endemic is the community? | How endemic is the community? Note: the given parameters may not precisely represent the community, but use them as a general guide for assessment. If you have more extensive knowledge about the community's endemism insert a comment explaining your score. |
| 20. Does this community harbor more endemic, highly disjunct, or evolutionary distinct species than other communities? | Please rate the approximate number of endemic, disjunct (at the species or generic level), or evolutionarily distinct flora and fauna that inhabits this community. The values below are relative, and your answer may depend on your taxonomic expertise (e.g., mammal specialist versus beetle specialists). |
| 21. Does this community harbor more endemic, highly disjunct, or evolutionary distinct species than other communities? | Please rate the approximate number of listed species (e.g., State or Federally Threatened or Endangered or species of special concern) that inhabit this community. |
| 22. Does this community type provide ecosystem services such as: storm surge attenuation, water cleansing, water storage, timber production, game or fisheries species production, rec, etc. | Does the community provide ecosystem services that can be quantified, such as stabilizing erosive shorelines, filtering water, serving as a carbon sink, providing clean air, etc., or is the community of economic value for commercial harvesting (timber, fisheries, hunting, etc.). Does the community serve a large ecotourism role (e.g., beaches). |

| SIVVA for Species Criteria | Criteria Descriptions and Instructions |
| --- | --- |
| *Vulnerability (Exposure + Sensitivity)* | |
| 1. Proportion of habitat inundated by or lost to SLR at X m by 2100 | Inundation scenario maps are provided; note, assume that habitat will not have time to migrate inland. |
| 2. Vulnerability to SLR through erosion in known range by substrate type/porosity inhabited | Score species by vulnerability to SLR in its current range according to substrate type or porosity. |
| 3.Vulnerability of current distribution or 'escape paths' to current or future barriers | Consult land-use maps. You should consider the red areas of the map (developed lands) as unsuitable habitat and a barrier to migration unless you know that this species does well in urban environments, and you can also consider natural barriers (e.g., open ocean surrounding islands) as blocking escape paths. |
| 4.Vulnerability due to dependence on a narrow temperature range | See map of 2100 temperature projections. |
| 5. Vulnerability due to dependence on a narrow range of annual or seasonal precipitation | See maps of 2100 annual and seasonal rainfall changes, consider these jointly in your assessment. |
| 6. Proportion of habitat already protected from development by a conservation area | Consult land-cover maps provided to you. |
| 7. Vulnerability to increased fragmentation of populations due to SLR, climate, and land-use | Will the changes in land use, sea level rise, and climate proposed in the Maps document likely fragment existing populations of this species? If 100% of their current distribution is likely to be inundated by SLR or otherwise rendered uninhabitable, you should put a score of 6. In other words, loss of habitat and presumed extirpation also fragments the species. |
| 8. Exposure and tolerance of increasing salinity due to SLR by 2100 | If the species lives in an area where there is likely to be saltwater encroachment (certainly any coastal county), what is its known tolerance? |
| 9. Exposure and tolerance of storm surge or runoff from impervious surfaces by 2100 | Using your knowledge of the species and its habitat and the inundation maps provided to score the vulnerability of the species to storm surge or runoff from heavy rain events under various scenarios |
| 10. Exposure and/or sensitivity to competition/ displacement/ disease/biotic interactions relating to SLR, land use and climate change | This includes all biotic interactions, including a species' prey, predators, commensals (pollinators), and competitors (including invasive species). This is also where you can account for displacement of upland species by people and other organisms retreating inland due to loss of coastal habitat. |
| 11. Vulnerability to synergistic effects of projections for 2100 SLR, development, and climate change | Based on the relevant maps, are there any synergistic effects in addition to those listed in the above criteria? |
| 12. Vulnerability to changes in a natural disturbance regime as a result of climate, land-use, or sea level changes by 2100 | Do changes in the future climate, land-use or sea level as described here result in changes to a natural disturbance regime (fire, seasonal storms, tree fall, etc.) that is critically important to this species? |
| *Adaptive Capacity* | |
| 1. Ability of species (not just individuals) to disperse away from 2100 threats- aka habitat tracking | Evaluate the dispersal capability of the species such that populations move around in space over short time periods relative to SLR and climate change. |
| 2. Phenotypic plasticity; including genetically controlled traits for which variation exists currently | This is where you can note that if a species inhabits areas with different climates and the species can probably handle variation in climate. |
| 3. Genetic diversity | Evaluate the state of the genetic diversity in this species relative to levels that are thought to have existed prior to human influence. |
| 4. Adaptive Rate (generation time, birth rate, fecundity) | Please rate the generation time, birth rate, fecundity and/or observed evolutionary rate of this species. |
| 5. Demographic capacity to adapt in situ or to migrate | Does species exist in populations that are large enough to adapt and/or migrate, or are populations too small and likely to "blink out" and go extinct? |
| 6. Adaptive capacity of life history traits that affect survival and recruitment in colonized areas | What is the ability of this species to colonize new habitats with just a few individuals? |
| *Conservation Value* | |
| 1. Level of endemism | Rate the degree to which the species is endemic to the region being evaluated. |
| 2. Disjunct from other populations outside of study area | Consider the relationship of the assessed range of the species with other populations of that species or other subspecies or closely-related members of the same genus. |
| 3. Keystone or Foundation Species | We will define a keystone or Foundation species as a species that has a disproportionately large effect on its environment relative to its abundance. Such species play a critical role in maintaining the structure of an ecological community, affecting many other organisms in an ecosystem and helping to determine the types and numbers of various other species in the community. |
| 4. Phylogenetic distinctiveness | Here we are giving value to species for which there are no other representatives of their lineage going back several millions of years. |
| 5. Ecosystem service provider or economically important species | Does the species provide ecosystem services that can be quantified, such as stabilizing erosive soils, or is the species of economic value that is harvested commercially, or does the species play a strong ecotourism role? |
| 6. Federal or State Listing | Please list the level of federal or state status (whichever is most elevated) |
| 7. Probability of recovery success | Following Joseph et al. (2009) and the idea that we should focus efforts towards saving species that are comparatively easy to save, rather than spending most of our funds on saving species that have a high probability of extinction. |
| *Information Availability* |  |
| 1. Published and/or unpublished literature or expert knowledge available | How much life history and conservation information does the community have on this species? |
| 2. Existing demographic or niche models available | Are there published models available? |
| 3. Population genetic data available | Are there good population genetic data available? |
| 4. Demonstrated response to sea level rise | Are their studies or models available of the species’ response to sea-level rise? |
| 5. Demonstrated response to climate change | Are there studies or models available of the species response to climate change? |
