# Supplementary material for: A multiscale natural community and species-level vulnerability assessment of the Gulf Coast, USA
Source: PLoS One. 2018 Jun 29;13(6):e0199844. doi: 10.1371/journal.pone.0199844 (PMC6025860; doi:10.1371/journal.pone.0199844)
Supplement: S1 File — (DOCX) [file pone.0199844.s002.docx]

S1 File. Descriptions of Natural Communities.

**Mangrove**

Mangrove is both a collection of trees and shrubs and a natural community found at the interface of land and sea in tropical and semi-tropical areas. There are four dominant mangrove species in the Gulf of Mexico: *Rhizophora mangle* (red mangrove), *Avicennia germinans* (black mangrove), *Laguncularia racemosa* (white mangrove), and *Conocarpus erectus* (button-mangrove or buttonwood). Black mangroves are the most tolerant of winter extremes and have the most northern range limit [1]. In general, the mangrove community is a colonizer of the intertidal zone and has adapted to changing salinities, inundated soils, shifting sediments, and dynamic coastlines. A possible exception is *Conocarpus erectus*, which does best on sheltered shorelines where freshwater flows and/or rainfall dilute seawater [2].

The northern extent and coverage of mangrove fluctuate in response to the duration, intensity, and frequency of extreme freeze events [3]. Rainfall and freshwater inflows also affect mangrove distribution, particularly in the Western Gulf. Mangrove distribution is restricted to the inter-tropical zone, between 30°N and 30°S latitudes and effectively follows the 20°C isotherm of seawater temperature, which depends on sea currents and can thus vary between winter and summer [4].

￼

Relatively mild winters over the past several decades have led to mangrove expansion into areas previously occupied by salt marsh plants [5]. Historically, high salinity and periodic freeze events have limited mangrove expansion, but changing climate patterns have resulted in mangroves displacing salt marshes in certain bays, such as Aransas Bay in Texas. However, when analyzed at a larger, regional level, this shift is not widespread. Instead, local, relative sea level rise is an important driver causing regional-level salt marsh loss.

Mangroves are a particularly sensitive ecosystem due to their narrow environmental tolerances, geographically restricted distribution, proximity to dense human populations in coastal zones, and their reliance on a few key framework species [4, 6]. Mangroves are vulnerable to changes in climatic conditions, especially freezing temperatures, rainfall, and the frequency of coastal storms [7]. Mangroves are able to keep pace with sea level rise through soil accretion as long as sea level rise remains below a certain threshold, about 12 cm per 100 years, but possibly up to 45 cm per 100 years [8]. They are also able to adapt to changing conditions through migration to new areas that become suitable due to inundation and increasing salinity levels as relative sea level rises. However, human use stressors such as shoreline modification, the loss of adjacent natural ecosystems to development, and the reduction of water quality can stress mangrove communities and make them more vulnerable to changing climate and sea levels.

Mangroves provide important ecosystem services to the regions in which they are found. They protect coasts from the effects of tropical storms and provide erosion control, water purification, and carbon sequestration. Many commercial fish species use mangrove roots as breeding and nursery habitat [9].

The focal species associated with mangroves for the purpose of this assessment is the roseate spoonbill.

**Roseate Spoonbill**

The roseate spoonbill (*Platalea ajaja*) is the only spoonbill that lives in the Western Hemisphere. It is a resident breeder in the Gulf of Mexico nesting along the coasts of Texas, Louisiana, and south Florida [10]. Outside of the breeding season, the roseate spoonbill can be found throughout the entire U.S. portion of the Gulf of Mexico coastline.

Roseate spoonbills feed on small fish and crustaceans. They are tactile foragers that feed most successfully when prey densities are high, which occurs when tides drop or drying wetlands concentrate prey into the deeper remaining pools [11]. Foraging habitat includes marine, estuarine, and freshwater sites such as tidal pools, estuarine and freshwater sloughs, mudflats, and mangrove-fringed creeks and can be farther inland than nesting sites [11]. Nesting is typically more restricted to mangrove islands and occasionally dredged-material islands, but also coastal swamp forests.

Roseate spoonbills reach sexual maturity at 3 – 5 years. Females typically lay 2 – 5 eggs that hatch after approximately 224 days [10]. Both parents incubate the nest. The young are able to fly as early as 6 weeks after hatching and typically have a 25-year lifespan (J. Lorenz pers. comm.).

**Tidal Emergent Marsh**

Tidal emergent marsh systems are a critical ecosystem along the Gulf Coast that support high levels of biodiversity and provide important ecosystem services, such as providing habitat for wildlife, fish, and other aquatic organisms and buffering coastal storms. The physiological tolerance of marsh species to salinity and inundation determine their abundance and often result in their use of the following three zones: salt marsh, brackish marsh, and fresh marsh [12]. These three zones are the focus of the GCVA. Causes of zonation possibly include succession [13], nutrient availability[14], and intra- and inter-specific competition [15], suggesting that the dynamics behind marsh zones require additional studies of physical, chemical, and biotic interactions.

Marsh elevation is a critical factor that determines not only the level of inundation, but also the ability of marsh species to survive and colonize new areas in response to rising sea levels. Tidal marshes may also be classified by relative elevation with respect to the tidal frame. Definitions on this basis include high, intermediate and low marsh, sometimes classified as regularly- flooded and irregularly-flooded. Relative elevation can interact with salinity to influence vegetation composition and growth. For example, high salt marshes are infrequently flooded by tides and dominated by herbaceous, emergent vegetation and forb-like dwarf shrubs due to evaporation-driven accumulation of salt in marsh soils. In contrast, intermediate and low salt marshes are more frequently flooded by tides and support more flood tolerant species.

Tidal marshes have been widely studied, providing a high understanding of the threats and stressors that most impact these ecosystems [12]. However, there are uncertainties in scientists’ ability to predict how tidal marshes and the species that depend upon them will respond to these stressors over time and in their ability to adapt to changing conditions. Marsh elevation is affected by coastal storms, which not only inundate marshes with saline waters, but also affect the amount of sediment either deposited or eroded from the shoreline [12]. Disturbance, either from coastal storms or human activities such as shoreline modification, can also increase vulnerability to the establishment of invasive species that can alter marsh community compositions or food webs [16]. Furthermore, invasive species possess qualities that may enable them to respond more positively to climate change than native species [17]. The ability of invasive species to exclude native species is not well understood [18], nor is it easy to identify potentially problematic species because there is not one unifying “invasive” characteristic [19].

The focal species associated with tidal emergent marsh for the purpose of this assessment are blue crab, clapper rail, mottled duck, and spotted seatrout.

**Blue Crab**

Blue crab (*Callinectes sapidus*) inhabits coastal waters from Massachusetts to the eastern coast of South America, including coastal waters of the Gulf of Mexico [20]. Shallow salt marsh and seagrass beds provide nursery habitat for juvenile crabs [21]. Mating occurs primarily in low-salinity waters of upper estuaries and lower portions of rivers. After mating, females will migrate to high-salinity waters in lower estuaries to the open Gulf to spawn [22, 23], while males remain in the creeks, rivers, and upper estuaries. Blue crab rarely moves from one estuarine system to another. Blue crab distribution is influenced by food and shelter availability, water temperature, and salinity [24].

Males mate for the first time during the third or fourth intermolt after maturing. Female crabs mate once in their lifetime, following the terminal molt to maturity, but store the sperm in seminal receptacles for multiple uses during a 1- to 2-year period [25, 26]. Fertilized eggs are extruded into a cohesive mass that contains 1 – 7 million eggs and is carried by the female for a ~10 day embryonic development period [27].

The blue crab is a valuable commercial species across its range and also has an important role in the structure and function of the estuary. In 2012, nearly 180 million pounds of hard blue crab were commercially landed nationally (a decrease of 9 percent from 2011), of which 53 million (a decrease of 3 percent) were landed in the Gulf Region (NOAA 2013). The blue crab is an important link in the estuarine food chain, serving as detritivores and scavengers throughout their range. They also act as both prey and consumers of plankton, invertebrates, fish, and other crabs. The blue crab is prey for several recreationally important fishes including spotted seatrout (*Cynoscion nebulosus*) and red drum (*Sciaenops ocellatus*).

**Clapper Rail**

Along the Gulf Coast, clapper rail (*Rallus crepitans*) distribution depends on the presence of tidal salt marsh and fiddler crab [28]. During low tide, rails move to exposed mudflats where they feed on fiddler crabs, their primary prey. Other food sources include minnows, insects, other birds’ eggs and, occasionally, small immobilized birds [29].

Nesting along the Gulf Coast begins in spring and extends to mid- to late summer [30]. Nests constructed of marsh grasses are built by males in higher areas of tidal marsh to avoid inundation during high tides. Females typically lay between 7 – 14 eggs, and the breeding pair takes turns incubating the nest for 20 – 24 days. Young are able to leave the nest soon after hatching and can fly by 63 – 70 days [30]. Clapper rails may have 1 – 2 broods per season. Following nesting, adults become flightless for several weeks as all flight feathers are dropped simultaneously. Almost contrary to this, and in addition to the fact that they are non-migratory, rails are excellent long-distance dispersers.

**Mottled Duck**

The mottled duck (*Anas fulvigula*) is a resident species that occurs along the Gulf Coast in two distinct populations. One inhabits peninsular Florida and the other is found from Alabama southwest to Tampico, Mexico [31]. Banding from thousands of birds indicates little to no exchange between the Florida and Western Gulf populations [32]. Mottled duck are a minor component of the overall waterfowl harvest in Texas and Louisiana. In the Western Gulf Coast, mottled duck use tidal fresh, intermediate, and brackish marshes as well as non-tidal freshwater wetlands and agricultural lands, notably rice and pasture. In peninsular Florida, they primarily use freshwater emergent wetlands and agricultural lands; however, they have also been found in artificially-created wetlands in urban and suburban areas.

Breeding pairs are formed from October through January. Breeding occurs from February through June. Nests are typically built in upland grass areas near wetlands and are often more than 1 km away from brood-rearing habitat. Males molt in July, while females molt in August and September after brood-rearing. Salinities of >9 ppt negatively affect mottled duckling survival [33]. Increased salinity through sea level rise could make these ducklings vulnerable.

**Spotted Seatrout**

Spotted seatrout (*Cynoscion nebulosus*) are common along the entire Gulf Coast but are most abundant off of south Texas, eastern Louisiana, Mississippi, and Alabama [34, 35]. They depend on estuaries for feeding, spawning, and nursery grounds. As top carnivores, they may help with the structure and function of estuarine communities. Spotted seatrout support valuable commercial and recreational fisheries.

Seagrass beds, where they occur, are the preferred habitat of post-larvae, juveniles, and adults; however, spotted seatrout may also occur abundantly near shell reefs, marshes, and submerged or emergent islands. Food availability in combination with a suitable salinity and temperature regime may also play an important role in the locations where they are found [36].

Spawning typically occurs at the end of the second or third year but has been reported as early as the end of the first year in both sexes. Peak spawning in the Gulf of Mexico occurs between late April and July. Egg estimates have ranged from 15,000 to 1.1 million, suggesting there may be variation among individuals or among estuaries [37].

**Oyster Reef**

Along the coast of the Gulf of Mexico, the eastern oyster (*Crassostrea virginica*), also known as the American oyster, is the dominant reef-building organism within the estuaries. Human activities, including altered river flows and over-harvest, have led to enormous losses of oyster reefs worldwide, with many reefs and populations being damaged beyond repair [38]. Oyster reefs are distributed throughout the Gulf of Mexico, and despite greater than 50 percent loss, this region is one of the few oyster ecosystems still in fair condition, making it possible to repair and restore oyster reefs to historical levels [38].

Along the northern Gulf Coast, oysters are sensitive to freshwater inflow into the estuaries. Increases in freshwater inflow lower salinity. If salinity decreases below 5 ppt for extended periods of time, oyster growth rates decrease, which may prevent spawning and possibly lead to increased mortality. In contrast, too little inflow may result in higher salinity, which can lead to increased predation pressure and disease prevalence. Numerous experimental and modeling results support these linkages. Beyond changing salinity, human activities involving alteration of the substrate may result in significant damage to oyster reefs through direct physical impacts [39]. This stressor and its effects are highly predictable.

Oysters and the reefs they form provide a variety of ecological services. Oysters improve water quality and water clarity through their filtration of water in the course of consuming algae; oysters filter up to 10 liters of water per gram of oyster tissue per hour [40]. They are also ecosystem engineers, forming reefs from the shells of oysters both living and dead, which then provide a hard substrate for oyster larvae to settle, continuing the reef building cycle. Oyster reefs also provide important habitat for many different species, alter currents, and reduce storm surge.

The focal species associated with oyster reefs for this project are eastern oyster, American oystercatcher, and red drum.

**Eastern Oyster**

Eastern oyster (*Crassostrea virginica*) is a commercially important species scattered throughout the bays and estuaries of the Gulf of Mexico. The eastern oyster is widely distributed in America from the Gulf of St. Lawrence, along the Atlantic coast of the United States, to the Gulf of Mexico, and through the Yucatan Peninsula to the West Indies and the coast of Brazil [41]. Oyster growth rate is dependent on temperature, salinity, and food supply. In the Gulf of Mexico, the optimum temperature range for oyster growth is from 20–30°C (Eastern Oyster Biological Review Team 2007). Eastern oysters are abundant in shallow saltwater bays, lagoons, and estuaries in water 2.5 – 7.5 m deep and water temperatures that can fluctuate between -2 and 32°C.

Oysters are filter feeders that feed primarily on phytoplankton and suspended detritus. When water temperatures exceed 35°C or drop below 5°C, the filtering rate slows and feed rate is affected. Oysters occur in areas with salinities between 0 and 40 ppt, with little growth occurring when salinities drop below 5 ppt (Eastern Oyster Biological Review Team 2007). As salinity levels increase, so do the threats from predators (such as Oyster Drill) and parasites such as *Perkinsus marinus*.

**American Oystercatcher**

Although there are two races of American oystercatcher (*Haematopus palliatus*) in the United States, only the eastern race (*Haematopus palliatus palliatus*), which occurs broadly from Nova Scotia to eastern Mexico, is found in the Gulf of Mexico. Within the Gulf of Mexico specifically, the American Oystercatcher Working Group (2012) identifies distribution from Lee County north to Bay County in Florida, with smaller populations of breeding birds in Alabama and Mississippi and west to Louisiana and Texas.

Along the Gulf Coast, American oystercatchers traditionally nest on barrier beaches, sandbars, shell islands, and marsh islands, but they have been found nesting on dredged-material islands and rooftops (Florida Fish and Wildlife Conservation Commission 2013). Nests, which are shallow depressions of scraped sand, are made in areas surrounded by water. After breeding season, roosting sites are typically utilized near feeding areas disconnected from the mainland. These birds often use shell rakes, which are aggregations of oyster and other shells found along the edges of marshy islands, for nesting and roosting (American Oystercatcher Working Group 2012). Their specialized bill makes them dependent on oysters and other bivalves as main sources of food.

American oystercatchers reach sexual maturity between 3 and 4 years of age and can live for more than 10 years [42]. Nesting season runs from February to August, and the female typically lays 2 – 4 eggs. Chicks are mobile within 24 hours of hatching but remain with parents for up to 6 months.

**Red Drum**

Red drum (*Sciaenops ocellatus*) is a highly mobile species found along the entire Gulf Coast [43]. Total estuarine area seems to affect their abundance [44]. Females can produce up to 2 million eggs and spawning peaks in September or October [45]. Larvae are carried by Gulf surface currents into estuarine nurseries. During this time, the fish are sensitive to poor water condition. Temperature and salinity affect larval development with larvae in warmer waters reaching juvenile stages faster than larvae in cooler waters [45]. Early cold spells reaching the Gulf can cause mass mortality. Larval fish also have little tolerance to low salinities.

Juveniles are found solely in the estuarine nursery and are more tolerant to low salinities than larvae. Tolerance to low salinity increases with age [36]. Juveniles prefer seagrass beds, shorelines, and shallow waters. They feed on shrimp, young blue crabs, copepods, gammarid amphipods, and fish. The red drum reaches sexual maturity around 3 – 6 years of age [45]. Adult drum are typically found within 5 miles of the Gulf shore. They are primarily bottom-feeders, but larger drum will feed on other fish. At this stage, the fish have the highest tolerance for a range of temperatures and salinities; however, they are sensitive to rapid and prolonged drops in water temperature.

Red drum was overfished for many years and is now closely regulated. Although a very popular game fish, commercial harvesting of red drum continues to be prohibited throughout the Gulf Coast states with the exception of Mississippi (Florida Fish and Wildlife Conservation Commission 2015). Red drum is vulnerable to degradation and destruction of estuarine habitat.

**Barrier Islands**

There is a total of 72 sand-rich barriers along the Gulf Coast that vary in character, composition, and level of human impact [46]. Although barrier islands have a range of geoenvironments, beaches and dunes are the focus of the GCVA and are identified across the Gulf Coast and by state [46].

Barrier islands are the first line of defense for protecting mainland coastal ecosystems from the direct effects of wind, waves, and storms. They also help maintain gradients between saline Gulf waters and inland estuarine systems [46]. Formed during the deceleration of sea level rise over the past 5,000 years, these islands persist from sand delivered from onshore sources and longshore transport. This migrating ecosystem is highly vulnerable to reductions in sand transport (through human modification), rising sea level, and tropical cyclones and storms, which can significantly change inundation regimes affecting the geomorphic structure of the barrier islands and the habitats they support. Long-term aerial imagery and sequential shoreline and bathymetric surveys along the barrier islands of the northern Gulf of Mexico have provided much of the understanding on geomorphic processes that dominate barrier island change and vulnerability.

The focal species associated with barrier islands for this project are black skimmer, Kemp’s ridley sea turtle, and Wilson’s plover.

**Black Skimmer**

The black skimmer (*Rynchops niger*) is a beach-nesting species found along the Atlantic coast from Massachusetts to southern Florida and west into the Gulf of Mexico through coastal south Texas [47]. Western populations also exist from California south through tropical South America.

Black skimmers nest in colonies on sparsely vegetated beaches, spoil islands, and occasionally gravel rooftops where nest success is poor [47]. Nests are made by creating slight depressions in the sand in which 3 – 4 eggs are laid [47].

Black skimmers forage for prey by dragging the lower bill through the water as they fly and closing the upper bill reflexively when prey is contacted (Florida Fish and Wildlife Conservation Commission 2013). Foraging sites include shallow waters offshore, freshwater bodies, estuaries, lagoons, and impoundments.

**Kemp’s Ridley Sea Turtle**

Kemp’s ridley (*Lepidochelys kempii*) is a highly migratory species of sea turtle that forages at sites throughout the Gulf of Mexico. The three main nesting regions are in in the state of Tamaulipas, Mexico; however, they do nest in the U.S., with the majority being in Texas with a few nests along the Florida panhandle [48]. Kemp’s ridley nesting occurs, typically in the daylight hours, in synchronized events called “arribada” (arrival) (National Wildlife Federation 2015). Kemp’s ridley occupies many areas within the Gulf of Mexico, with their primary habitat being the nearshore and inshore waters. The Kemp’s ridley reaches maturity at 10 – 15 years of age. Once they have hatched, males spend their entire lives at sea, while females leave the ocean only to lay eggs. Female turtles congregate in shallow water and all emerge at once to lay eggs on the beach (the arribada). On average, females lay 1 – 4 clutches of eggs every two years. Each clutch can have between 50 and 130 eggs [49]. When female hatchlings reach maturity, they return to the site where they hatched to lay their own eggs, but sometimes move to other beaches. Adults mainly occupy neritic habitats that have muddy or sandy bottoms where prey can be found. Their diet consists mainly of swimming crabs, but they also eat jellyfish, fish, and mollusks [49].

Kemp’s ridley are the world’s most endangered sea turtle due to overharvesting of eggs and loss of juveniles and adults to commercial fishing activities in the mid-1900s [50]. From 2009 to 2015, there has been a 40% decline in Kemp’s ridley nests; the cause of this decline is still being researched.

**Wilson’s Plover**

Wilson’s plover (*Charadrius wilsonia*) is a medium-sized shorebird found primarily in coastal ecosystems. It can nest in a variety of beach microhabitats from barren to densely vegetated substrates above the high-tide line [51]. They are visual feeders that prefer fiddler crabs and other small crustaceans found on exposed mudflats. Within the U.S. portion of the Gulf of Mexico, Wilson’s plover breeds across the region from Florida to south Texas and winters primarily in northeast and central Florida, west Louisiana, and Texas [52].

The males build nests by making multiple scrapes in the sand of sparsely vegetated saline areas such as beaches above high tide, dune areas, and the edges of lagoons. Females lay 2 – 4 eggs, and parents share incubation for approximately 28 days [52, 53]. If a nest fails, re-nesting can occur with 5 – 13 days [53]. Chicks are mobile shortly after hatching and use nearby vegetation to hide.
