# Supplementary material for: A multiscale natural community and species-level vulnerability assessment of the Gulf Coast, USA
Source: PLoS One. 2018 Jun 29;13(6):e0199844. doi: 10.1371/journal.pone.0199844 (PMC6025860; doi:10.1371/journal.pone.0199844)

S1 Fig. Climate summaries and seasonal averages.

Laguna Madre


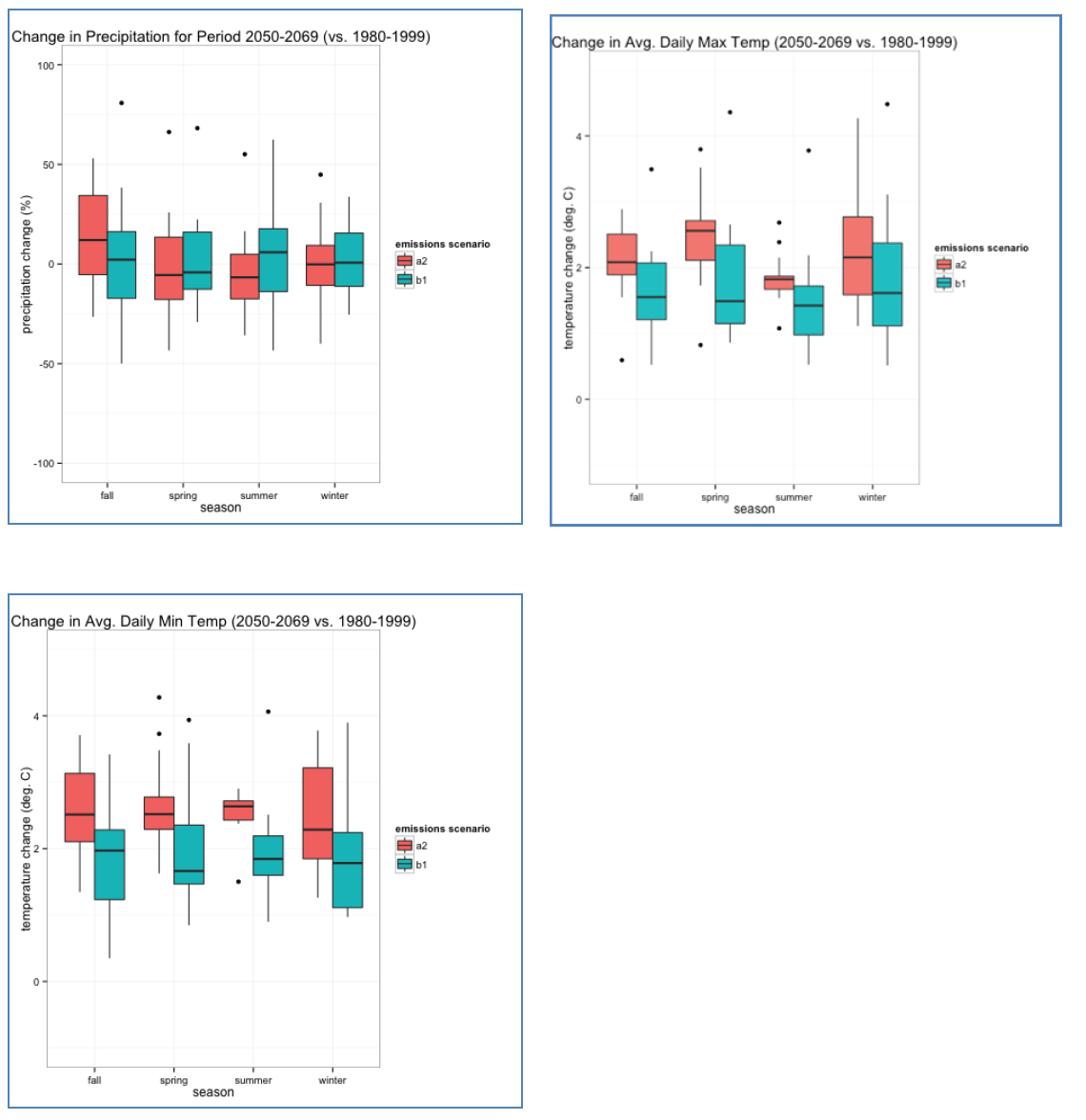


Western Gulf Coastal Plain


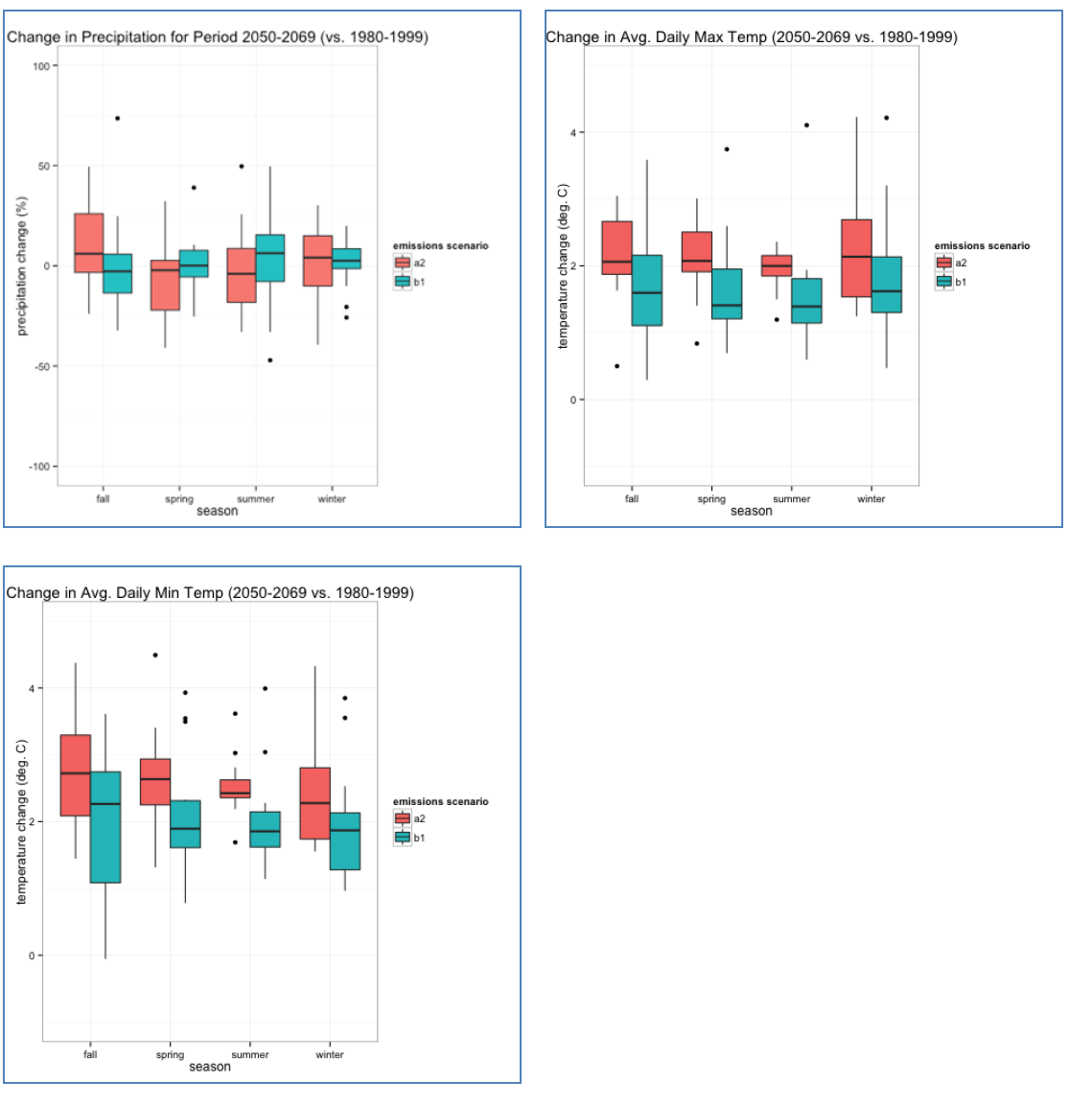


Mississippi Alluvial Plain


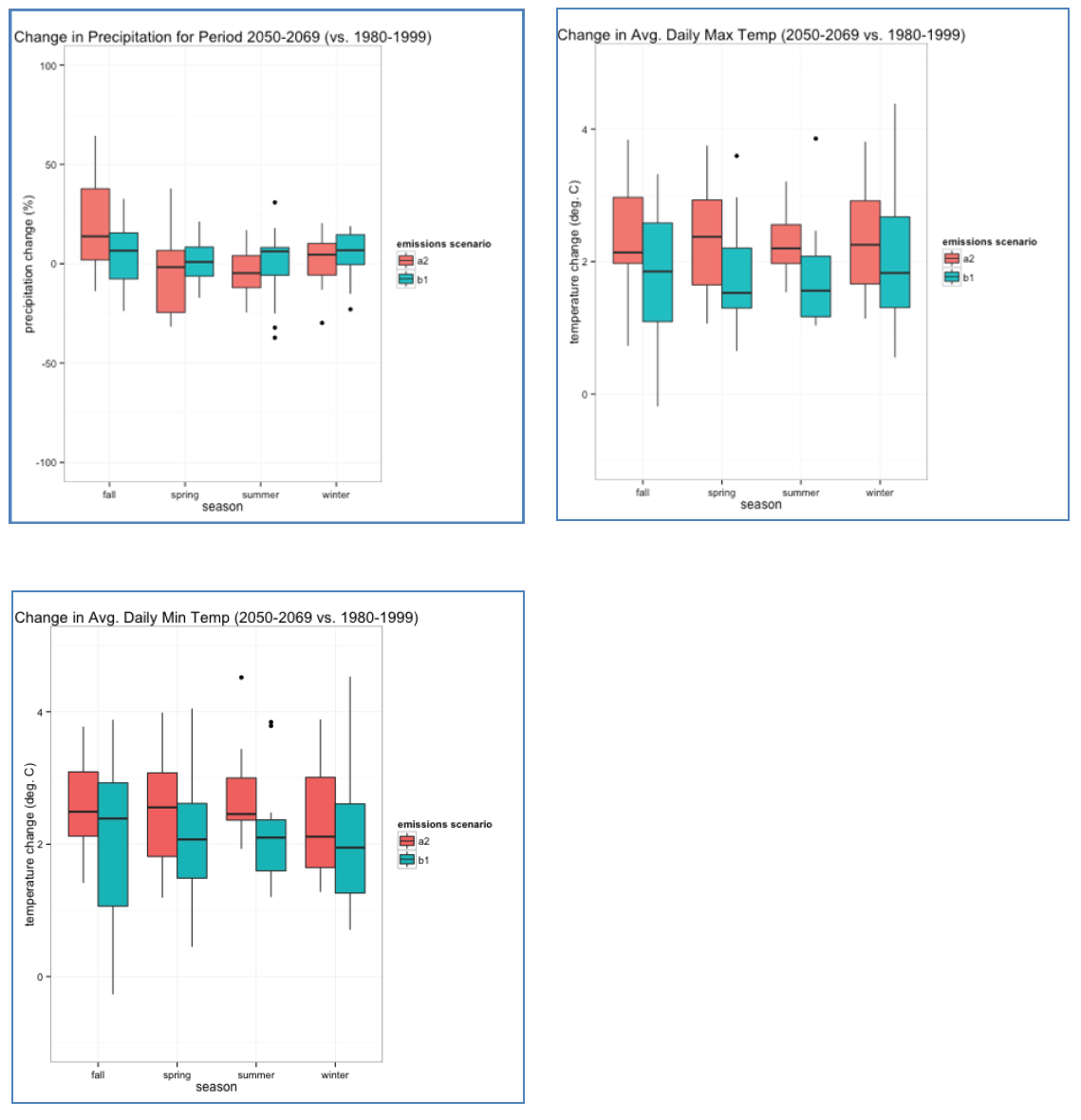


Southern Coastal Plain


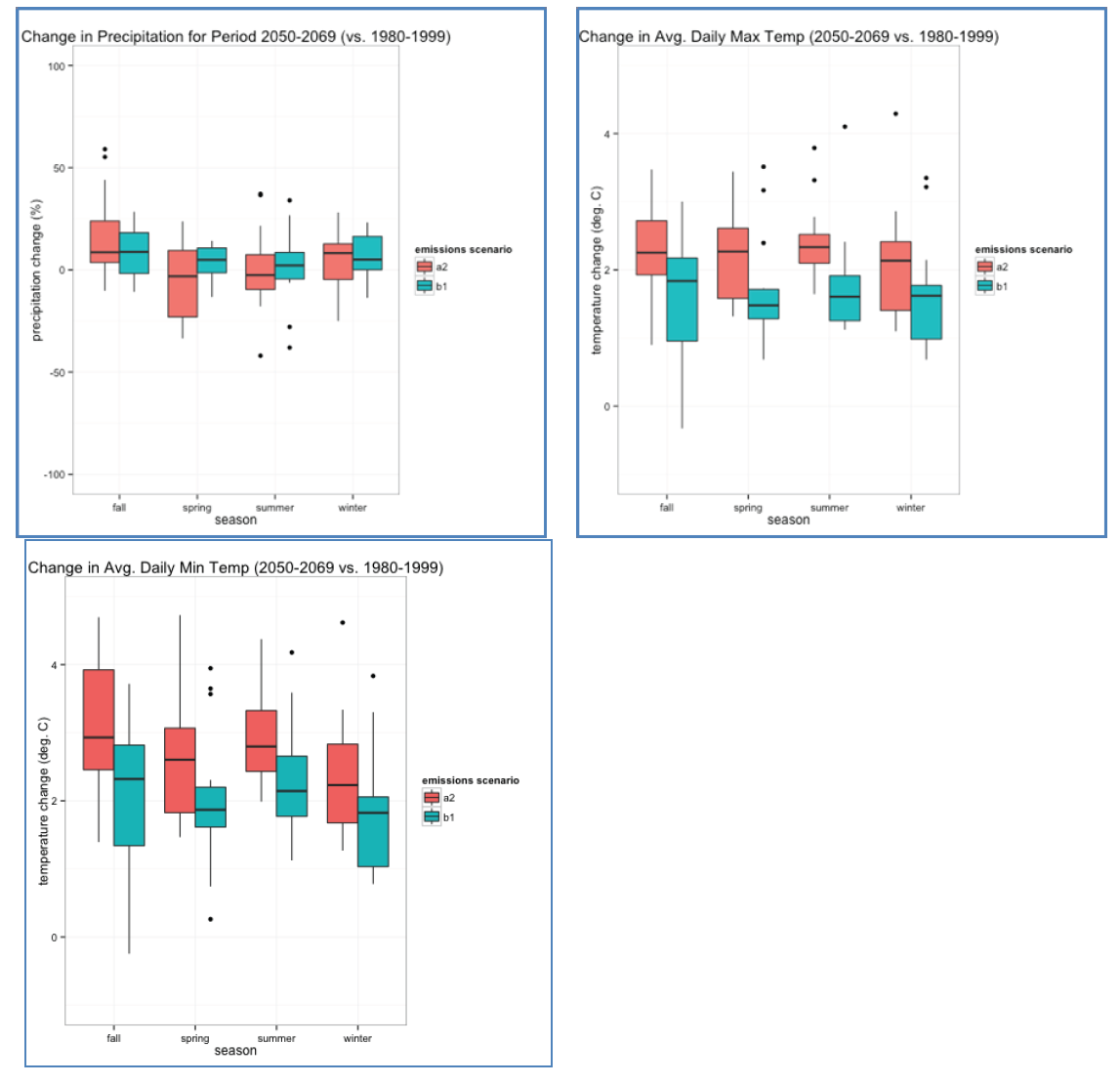


Central Florida Coastal Plain


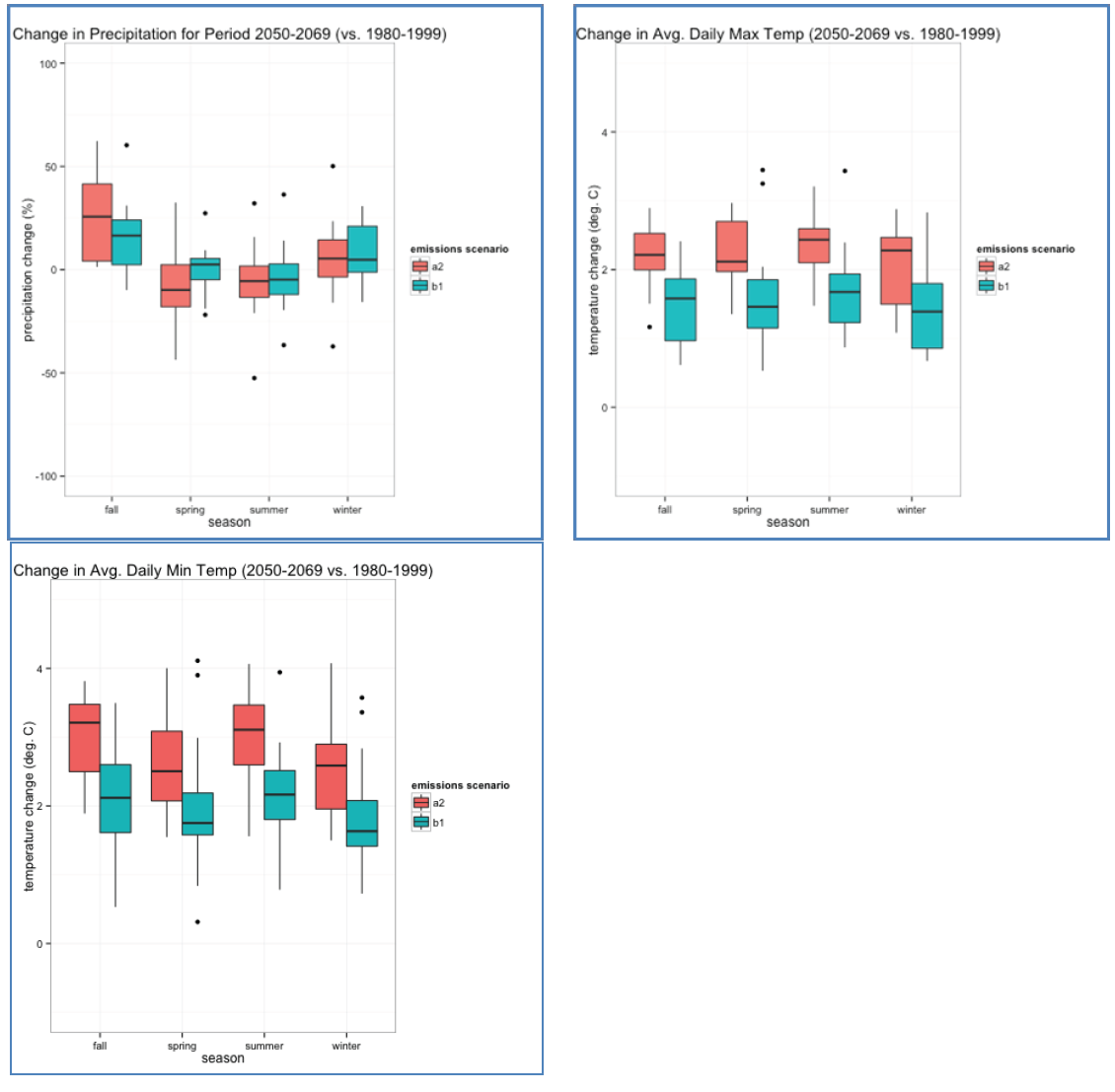


Southern Florida Coastal Plain


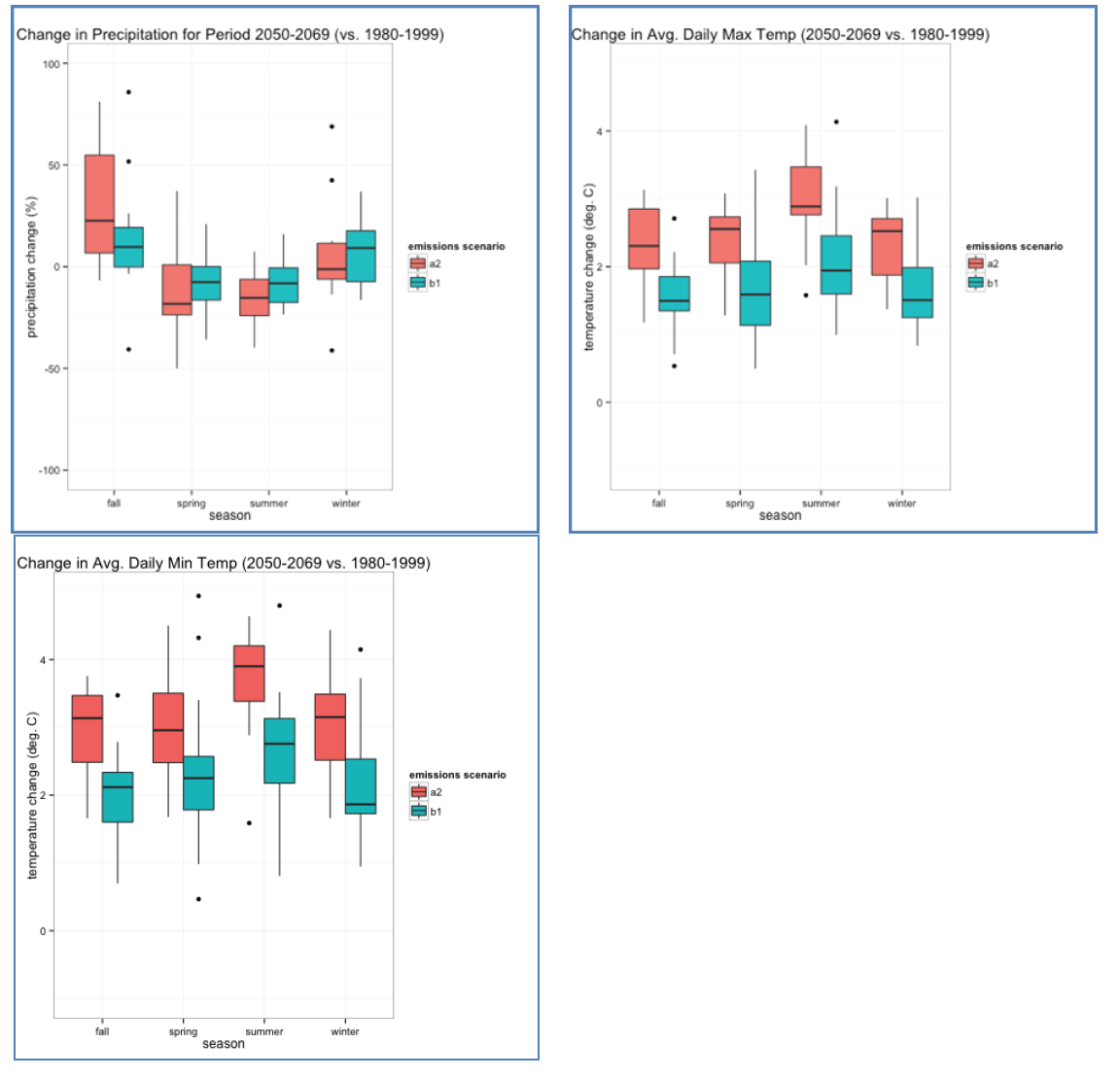


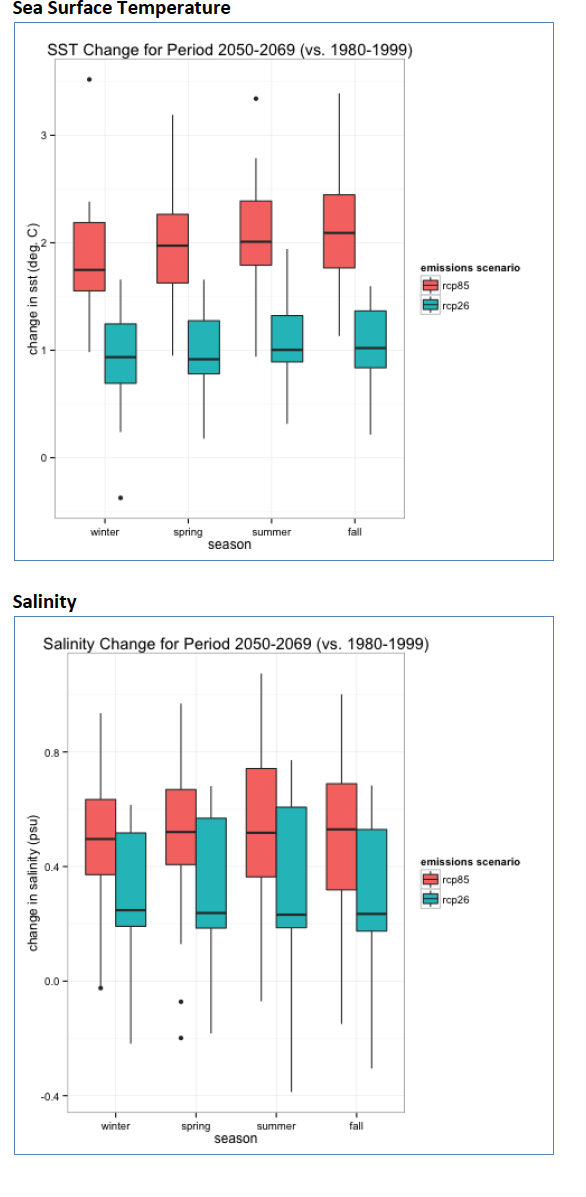

Supplement: S1 Fig — (DOCX) [file pone.0199844.s003.docx]
