# Supplementary material for: A multiscale natural community and species-level vulnerability assessment of the Gulf Coast, USA
Source: PLoS One. 2018 Jun 29;13(6):e0199844. doi: 10.1371/journal.pone.0199844 (PMC6025860; doi:10.1371/journal.pone.0199844)
Supplement: S2 File — (DOCX) [file pone.0199844.s004.docx]

S2 File. Assessor Variation.

Figures in this file illustrate variation in expert opinion. For the SIVVA NatCom assessment, the SIVVA scores presented below are the mean of the Ecosystem Status, Vulnerability, and Conservation Value modules. In the SIVVA for Species assessment SIVVA scores presented below are the mean of all four modules (Vulnerability, Adaptive Capacity, Conservation Value, and Information Availability). In both cases, the dotted line is the average SIVVA score for all ecosystems and species. The pair of lines closest to the dotted line are one standard deviation above and below, and the next set of lines are two standard deviations above and below the mean (which is equivalent to the 95% confidence interval for our purposes). Scores given by experts are averaged across subregions, climate scenarios, and species or habitats.
