# Supplementary material for: A multiscale natural community and species-level vulnerability assessment of the Gulf Coast, USA
Source: PLoS One. 2018 Jun 29;13(6):e0199844. doi: 10.1371/journal.pone.0199844 (PMC6025860; doi:10.1371/journal.pone.0199844)
Supplement: S3 File — (DOCX) [file pone.0199844.s005.docx]

S3 File. Results of vulnerabilities for natural communities and focal species.

**Mangrove**

Ecosystem Status

The largest mangrove areas occur in the Central Florida Coastal Plain and Southern Florida Coastal Plain with approximately 554,515 acres combined. Mangroves have been mapped in Texas and Louisiana, but this has occurred sporadically so acreage is hard to determine. Localized accounts of mangrove expansion have been documented in Tampa Bay, Florida [54], Louisiana [20], and the Ten Thousand Islands region of Florida [55-58]. Regional-level mangrove expansion along the Texas coast has also been documented [5].

Potential Impact

Generalizing climate impacts on mangroves is difficult due to the variety of environmental settings in which mangroves occur [59]. This may explain some of the variation in expert opinion that occurred within a given subregion, especially regarding mangrove loss to sea level rise in Florida. Within any of the subregions, the range of mangroves lost to sea level rise ranged from nearly complete inundation to a possible increase in mangrove coverage. As noted under Ecosystem Status, mangrove area outside of the Central and Southern Florida Coastal Plains is limited, and the vegetation type was not explicitly included in SLAMM for the remaining subregions. Therefore, some of the variation in expert judgment is likely due to the lack of modeling for mangrove. Experts noted that assumptions in SLAMM are based on salt marsh, so additional resources should be used when assessing mangrove vulnerability to sea level rise. Other factors that should be considered include subsidence, species composition, salinity, and hydrologic connectivity, among other factors[60]. The ability to keep pace with relative sea level rise will ultimately depend on the mangroves’ ability to accrete soil and build its elevation [59]. In the Everglades region, saltwater intrusion into freshwater marsh and swamps will likely allow for the expansion of mangroves [59].

Expert judgment also varied on the impacts of how changes to disturbance regimes will influence mangroves. Experts were not given a list of disturbances to assess, so variation reflects what disturbances each individual considered. Disturbances that may impact mangroves include the frequency and intensity of tropical storms and severe freeze events, as well as changes in CO^2^ levels. Tropical storm events can negatively impact mangroves through outright destruction and erosion of sediments, counteracting any gain in mangroves [61]. Mangrove expansion into northern parts of the Gulf is currently limited by the frequency, duration, and intensity of extreme winter events (i.e. freezing air temperatures). For the Southeastern United States, found that mangrove forests are not likely to be present in areas where 30-year minimum air temperatures fall below -8.9°C, and mangrove forests are not likely to be dominant in areas where 30-year minimum air temperatures fall below -7°C [3]. Should the frequency, duration and/or extreme winter air temperature events decrease, mangrove forests in northern areas of the Gulf of Mexico are expected to expand at the expense of salt marsh. Changes in CO^2^ concentrations can enhance the growth of some mangrove species, but responses are often confounded by other factors such as salinity, nutrient availability, and water-use efficiency [7]. Elevated CO^2^ may enhance mangroves’ ability to supplant marsh especially when competition and herbivory are low [62].

Experts did agree across and within subregions that hydrologic changes were likely to have a negative impact on mangroves. Land use change, dams, pumping of groundwater, and other human activities can affect pollution and nutrient levels in freshwater, increase salinity of water reaching the system, and alter the sediment budget which is critical for maintaining mangrove elevation [4]. Changes in precipitation may further alter freshwater availability which is especially critical in freshwater-limited areas (e.g., where rainfall is less than 1 m per year such as south and central Texas) [63].

Constraints on range expansion were noted in the Laguna Madre, Central Florida Coastal Plain, and Southern Florida Coastal Plain. In the Laguna Madre subregion, assessors noted that mangrove migration could be limited by the hypersaline conditions. In southern Florida, projected human population growth is among the highest rates nationally, including those neighboring the northern Everglades [59]. While direct destruction of mangroves is not likely to occur due to protection, human population growth and the subsequent development of areas that are not mangroves could limit its migration.

Vulnerability

Mangroves were judged to be highly vulnerable in the Laguna Madre, Central Florida Coastal Plain, and Southern Florida Coastal Plain; and moderately vulnerable in the Western Gulf Coastal Plain, Mississippi Alluvial Plain, and Southern Coastal Plain. Mangrove expansion has been documented in Texas, Louisiana, and Florida; however, future expansion will be dependent on the ability of mangroves to keep pace with sea level rise. The high vulnerability scores reflect mangrove loss based on SLAMM estimates and constraints on range shifts.

The vulnerability of mangroves was calculated by averaging the scores from the Ecosystem Status and Potential Impact modules. Scores in the Ecosystem Status and Potential Impact modules were averaged across experts.

**Roseate Spoonbill**

Potential Impact (Exposure + Sensitivity)

Roseate spoonbill primarily nests on mangrove dominated islands in the Central Florida Coastal Plain and Southern Florida Coastal Plain. In the remaining subregions, experts noted scrub-shrub habitats in estuarine and palustrine emergent wetlands and cypress trees in palustrine forested wetlands are used. In each subregion an estimated 25 – 50% of roseate spoonbill habitat may be inundated by 0.41 – 0.82 m of sea level rise. However, there is a great deal of uncertainty regarding sea level rise impacts to colonial waterbird habitat. It was noted that there could be substantial loss to currently used sites, but new habitat may be created as marshes and large islands are fragmented. Large islands are currently unsuitable for roseate spoonbills due to the presence of mammalian predators [64]. The smaller, fragmented islands might be too small to support mammalian predators and thus suitable for nesting. A projected increase in mangrove coverage could also provide nesting substrate.

While loss of nesting habitat may not be an issue for roseate spoonbill, foraging habitat could be impacted. Roseate spoonbills forage at shallow marine, estuarine, and freshwater sites, with most foraging occurring in seasonally flooded wetlands and shallow creeks [65]. Intermediate salinities are needed to support prey at these foraging sites; saltwater intrusion, management practices that affect the hydrologic regime, and tropical storm activity could change the salinity levels [11]. If prey numbers decline or prey is dispersed, foraging becomes less efficient, and spoonbills can suffer a decrease in nest success [66]. While it is difficult to determine how the combined effects of climate, sea level rise, and land use change will impact roseate spoonbills due to limited information availability, most experts felt that combined effects will have negative consequences for the species.

Adaptive Capacity

Assessing the adaptive capacity of species is more subjective than potential impacts because life history and adaptability data are often more limited. Lack of adaptive capacity was rated highest in the Southern Coastal Plain due to small population sizes, the inability to colonize new areas, and the lack of phenotypic variation expressed by spoonbills. Roseate spoonbills are broadly distributed from South America (east of the Andes) to coastal Central America, the Caribbean, and the Gulf of Mexico [10]. Because they experience a range of environmental conditions, roseate spoonbill may be able to cope with projected changes. The species is also highly mobile with the potential to disperse away from threats; however, there must be suitable habitat available. Although the bird’s ability to colonize new areas is generally uncertain, one assessor noted evidence supporting their ability to colonize new areas given that their distribution has changed in Louisiana over the last 50 years, expanding from southwest to southeast Louisiana and north past Interstate 10. It has not been possible to estimate the number of birds involved in these expansions. Roseate spoonbill reaches maturity between 3 – 5 years of age and produces 1 – 3 chicks per nesting cycle. Species that have shorter reproduction times and high productivity are typically thought to be more adaptive [67].

Vulnerability

Roseate spoonbill was judged to be most vulnerable in the Southern Coastal Plain and Central Florida Coastal Plain. This is due to the increased coastal development in these subregions and the associated water management impacts that accompany population growth. The overall adaptive capacity module received higher scores (i.e. less adaptive capacity) by experts in these subregions, which also contributed to the higher vulnerability score. In the Laguna Madre, Western Gulf Coastal Plain, and Mississippi Alluvial Plain, coastal development is less of an issue, and the score for the adaptive capacity module was lower in these subregions. Consequently, roseate spoonbill vulnerability was lower in these areas. Gulf-wide threats include changes to biotic interactions (specifically prey), loss of habitat to sea level rise and erosion, and storm surge. For each subregion, the vulnerability of roseate spoonbill was calculated by averaging the scores from the Potential Impact (exposure + sensitivity) and Adaptive Capacity modules. Scores in the Potential Impact and Adaptive Capacity modules were averaged across experts.

**Tidal Emergent Marsh**

Ecosystem Status

Across the Gulf, marsh acreage has been declining. Between 1998 and 2004, about 49,670 acres of freshwater emergent marsh and 44,090 acres of estuarine (brackish and salt) emergent marsh were lost along the Gulf Coast [68]. The highest freshwater marsh loss occurred from central Texas to Apalachicola, Florida. Loss of estuarine marsh was most noticeable in Texas, Louisiana, and Mississippi. NOAA (2010) indicated that wetlands across the Gulf of Mexico were primarily lost to open water (48%) and development (28%).

Potential Impact

Sea level rise and erosion will result in the direct loss of marsh across the Gulf. However, SLAMM projections also show marsh migration inland into new areas, a phenomenon that is exacerbated as freshwater and brackish marsh become more suitable for salt marsh. Where shifts do occur, there may be a change in ecosystem function.

Direct loss to urban development was not judged to be a direct threat to marsh in most subregions. However, in the Southern Coastal Plain, experts felt there could be some areas where development reduces tidal emergent marsh by 50 – 79%. Urbanization could also limit the ability for marsh to migrate inland.

Tidal emergent marsh in all subregions is likely to experience fragmenting. It was noted by experts that fragmentation is particularly severe in the Mississippi Alluvial Plain where construction of the federal Mississippi Rivers and Tributaries levee project has substantially reduced sediment and freshwater delivery to the nearby wetlands. Subsequent work on freshwater diversions has attempted to reverse this by restoring the supply of sediment needed to build land in the river deltas.

Experts across all subregions noted that tidal emergent marsh is already suffering from changes to the disturbance regime by way of altered river flooding cycles that have resulted in reduced sediment loading and freshwater inflow. Future changes to other disturbance regimes, such as tropical storm frequency and intensity and winter minimum temperature changes, will exacerbate marsh loss. Increased winter minimum temperatures may allow for the expansion of black mangrove into areas currently occupied by marsh. This is currently happening in stands of *Spartina* in the Laguna Madre and the West Gulf Coastal Plain. Potential increases in the frequency and intensity of hurricanes can cause rapid decreases in marsh area due to the complete submergence of marsh from storm surge and the breakdown of marsh from pounding surf [69].

Invasive species such as *Hydrilla*, *Salvinia*, water hyacinth, and nutria can negatively impact marsh systems, especially in freshwater marsh. In the Central Florida Coastal Plain, experts commented that invasive vegetation (mainly Brazilian pepper and Australian pines) encroach upon landward boundaries of salt marsh habitat, restricting landward migration in response to sea level rise.

Vulnerability

The vulnerability of tidal emergent marsh is high across the entire Gulf coast, except in the Southern Florida coastal plain where it is very high. Sea level rise, fragmentation of the ecosystem, altered hydrology, and constraints on range shift were typically judged to be the most serious threats across all subregions. In the Southern Florida Coastal Plain, these threats were judged to have severe negative impacts on marsh as compared to the other subregions.

For each subregion, the vulnerability of tidal emergent marsh was calculated by averaging the scores from the Ecosystem Status and Potential Impact modules. Scores in the Ecosystem Status and Potential Impact modules were averaged across experts.

**Blue Crab**

Potential Impact (Exposure + Sensitivity)

Blue crab is not likely to be negatively affected by climate change, sea level rise, and land use change. As noted in the tidal marsh ecosystem assessment, marsh fragmentation is a major concern; however, blue crab uses marsh edge, which will increase with marsh fragmentation [70]. While there is the potential for “too much” edge, that threshold is currently unknown. As some marsh areas are converted to open water, blue crab may use submerged structures, such as oyster reefs, for cover. It was noted by experts that should salinity and SST change within the estuary, blue crab would potentially shift geographically to new areas where conditions become suitable.

Adaptive Capacity

Blue crab was judged by experts to have the highest adaptive capacity of the 11 species assessed. Experts indicated the blue crab has high mobility, is widely distributed from North America to South America, and exists in large populations. They have high genetic diversity, with the larval population mixing near the continental shelf [71]. Females lay up to 7 million eggs per brood [27]. Larvae can be transported for distances of 300 km or more, which enhances their ability to colonize new areas [70]. These characteristics support the ability to adapt to new environmental conditions by either migrating away from threats or potentially adapting to new conditions.

Vulnerability

Blue crab vulnerability is low across all subregions. Their mobility and ability to tolerate a range of conditions are two characteristics that may be especially helpful in adapting to future conditions. Blue crab may also benefit from an increase in marsh edge [72].

For each subregion, the vulnerability of blue crab was calculated by averaging the scores from the Potential Impact (exposure + sensitivity) and Adaptive Capacity modules. Scores in the Potential Impact and Adaptive Capacity modules were averaged across experts.

**Clapper Rail**

Potential Impact (Exposure + Sensitivity)

Loss of tidal emergent marsh habitat was judged to be more severe in the Laguna Madre, Western Gulf Coastal Plain, Mississippi Alluvial Plain, and Southern Florida Coastal Plain. Experts noted that SLAMM models estimated that marsh accretion rates will keep up with sea level rise in the Southern Coastal Plain and the Central Florida Coastal Plain, but they questioned whether that was actually the case. In the Laguna Madre, Mississippi Alluvial Plain, and Southern Florida Coastal Plain, marsh fragmentation may negatively affect clapper rail’s dispersive potential and population connectivity.

Across the Gulf, a predicted increase in hurricane frequency and the associated storm surge pose a threat to the species. Experts commented that although adults may be able to survive storm surge conditions, nests that are located low on the vegetation in salt marsh are easily flooded. It was also noted that although immediate impacts may be negative, clapper rails might benefit from the ecological release from predation following storm events.

Some experts felt that potential changes in biotic interactions may negatively impact clapper rail. Reasons provided by the experts included increased encounters with predators as rails are pushed to their habitat limits; decreased availability of fiddler crabs, their main food source; and increased encounters with humans.

Adaptive Capacity

Compared to other birds that were assessed, the clapper rail was judged to be less mobile. Assessors noted that while clapper rails possess the ability to migrate away from threats, they tend not to make large movements. Potential movement would also be limited by the availability of habitat. With the exception of the Laguna Madre and Southern Florida Coastal Plain, the clapper rail exists in large populations, which may enhance its ability to adapt to changes. However, it was noted that they are also strictly tied to their habitat, so migration may not be possible if habitat is not available. Clapper rails show some regional variation in phenotypic traits. Assessors noted that while there is not much variation in habitat choice, bill lengths vary across the range and may allow for a prey shift. The clapper rail produces multiple eggs yearly; typically one of the young survives every year to every other year.

Vulnerability

Clapper rail vulnerability varies from moderate to high. In the Laguna Madre, there are few clapper rails because tidal emergent marsh is limited in this subregion. Consequently, clapper rails may be more susceptible to projected threats and population fragmentation in this subregion. In the Southern Florida Coastal Plain, a subspecies of clapper rail occurs. Gulf-wide threats to clapper rail include loss of habitat to erosion and increased storm surge and hurricane frequency.

For each subregion, the vulnerability of clapper rail was calculated by averaging the scores from the Potential Impact (exposure + sensitivity) and Adaptive Capacity modules. Scores in the Potential Impact and Adaptive Capacity modules were averaged across experts.

**Mottled Duck**

Potential Impact (Exposure + Sensitivity)

Tidal emergent marsh loss to sea level rise was judged to be an issue for mottled duck in the Western Gulf Coastal Plain, Mississippi Alluvial Plain, and Southern Coastal Plain. The marsh is already eroding in many of these areas, and sea level rise will compound the problem. In the other subregions, it was noted that mottled duck utilizes other habitat types more frequently. In Florida, most of the population is supported by freshwater emergent habitats, which may be lost as salinity increases because of saltwater intrusion from sea level rise. In the Laguna Madre, information is limited regarding mottled duck nesting, but they likely use inland palustrine wetlands.

Across most of the subregions, precipitation changes will not likely impact mottled duck. However, experts note that the Laguna Madre subregion is semi-arid, and even a small decrease in precipitation could affect the availability of freshwater wetlands.

Although there is uncertainty regarding the synergistic effects of sea level rise, climate change, and land use change on mottled duck due to limited information availability, assessors agreed mottled duck will likely experience negative impacts due to interactions of these three drivers. Movement of humans away from the coast to inland peninsular Florida may have negative impacts on freshwater emergent wetlands because of development, pollution, and water usage. Assessors noted that introduction of the domestic mallard could have negative effects on mottled duck through hybridization; an issue that is already occurring in Florida (Florida Fish and Wildlife Conservation Commission 2014).

Adaptive Capacity

Mottled ducks are highly mobile and utilize a variety of different habitats, so they will likely be able to disperse away from threats. However, suitable breeding habitat is found only along the Gulf Coast so the population is somewhat limited in its dispersibility. Assessors noted some regional variation in phenotypes. For example, in Florida mottled duck has adapted to urban landscapes, but this has not occurred in all of the subregions. Mottled duck may be able to cope with projected environmental changes, but there is uncertainty regarding how population size will be influenced. Experts estimated mottled duck to have intermediate to high genetic diversity. Species with high genetic diversity may possess some heritable traits that will allow them to cope with projected change [73].

There are two populations of mottled duck along the Gulf Coast. One population is a resident of peninsular Florida with an estimated 30,000 individuals, and the other population is resident from Alabama westward to Mexico. This population is estimated at 630,000 individuals (North American Waterfowl Management Plan, Plan Committee 2004). Expert responses varied on the ability of mottled duck to colonize new areas. Some felt only a few individuals would be capable of starting a new population while others felt repeated introductions with dozens of individuals would be necessary.

Vulnerability

Mottled duck was judged to be moderately vulnerable across the Gulf. In general, assessors thought that although the species may experience some negative impacts associated with climate and land use change, the population will probably not be strongly affected. The mottled duck’s demonstrated ability to adapt to a variety of habitats will likely contribute to the species’ ability to adjust to change.

For each subregion, the vulnerability of mottled duck was calculated by averaging the scores from the Potential Impact (exposure + sensitivity) and Adaptive Capacity modules. Scores in the Potential Impact and Adaptive Capacity modules were averaged across experts.

**Spotted Seatrout**

Potential Impact (Exposure + Sensitivity)

The GCVA associated spotted seatrout with tidal emergent marsh; however, many experts noted the fish’s use of submerged aquatic vegetation (SAV) and open water as habitat. SAV and open water may actually increase as a result of sea level rise. Marsh edge is also likely to increase as marsh becomes fragmented in response to sea level rise.

Projected temperature increases could potentially exceed thermal maximums for spotted seatrout. Optimum temperature for eggs and larvae was reported to be 28°C, but the same study predicted 100% survival up to 32.7°C [74].

Very little spotted seatrout habitat is protected by conservation areas. Spotted seatrout are a popular recreational fishery. Consequently, lack of protected habitat free from fishing pressure may negatively affect the fish [75].

Adaptive Capacity

The ability of spotted seatrout to disperse away from future threats varied. In the Laguna Madre, Central Florida Coastal Plain, and Southern Florida Coastal Plain, experts felt the species could disperse from threats more than experts in the Western Gulf Coastal Plain, Mississippi Alluvial Plain, and Southern Coastal Plain. In Louisiana, there is some evidence that spotted seatrout movement varies by sex [76]. Females exhibit estuarine fidelity while males will leave their natal estuary and spawn in another area. Assessors think that spotted seatrout exhibit high genetic diversity, which can improve fitness. Most spotted seatrout reach maturity between years 2 and 3 [77]. Depending on size, a female can produce between 15,000 and 1,100,000 eggs. Assessors think that spotted seatrout show some regional variation in phenotypes, which will allow them to adapt to projected changes.

Vulnerability

Vulnerability of spotted seatrout to future conditions ranged from low in the Laguna Madre, Central Florida Coastal Plain, and Southern Florida Coastal Plain to moderate in the Western Gulf Coastal Plain, Mississippi Alluvial Plain, and Southern Coastal Plain. In subregions with moderate vulnerability, loss of habitat to sea level rise and erosion were judged to be more severe. Consequently, the limited ability of spotted seatrout to migrate away from threats in those subregions also increased vulnerability.

For each subregion, the vulnerability of spotted seatrout was calculated by averaging the scores from the Potential Impact (exposure + sensitivity) and Adaptive Capacity modules. Scores in the Potential Impact and Adaptive Capacity modules were averaged across experts.

**Oyster Reef**

Ecosystem Status

The percentage of oyster reefs considered to be functionally extinct in the Gulf of Mexico was recently evaluated [38]. In the Laguna Madre, West Gulf Coastal Plain, Mississippi Alluvial Plain, and Southern Coastal Plain, they estimated 50 – 89% of oyster reefs are functionally extinct. In the Central Florida Coastal Plain and Southern Florida Coastal Plain, oyster reef loss was estimated to be 90 – 99%. No estimates for Louisiana were given due to limited historic data.

Oyster reef function in the Central and Southern Florida Coastal Plain was classified as poor [38]. Evidence indicates that the fishery is collapsing or collapsed, but the reefs still remain. In the remaining subregions, oyster reef function was classified as fair, abundance indicators were below 50% of historical figures, or records indicated greater than 50% loss in reefs, yet there was evidence of significant remaining reefs. Despite these declines, oyster reefs from the northern Gulf of Mexico still were estimated to provide average annual catch of over 50,000 tons of wild native oysters, the largest quantity of any region in the world [38].

Potential Impact

Changes to the natural disturbance regime resulting from projected 2060 changes in climate, land use, and sea level will negatively affect oyster reefs, causing moderate decreases in extent and/or ecosystem function. Salinity changes resulting from altered weather patterns are key, as are timing of increased or decreased precipitation. Small increases in sea surface temperature can also affect oyster growth and survival, largely through the interactive effects of low salinities with high temperatures, which can lead to increased mortality of individual oysters [78].

Changes in hydrology that affect salinity could negatively impact oyster reefs. Oysters that exist after marsh loss may experience ‘flashy’ hydrological conditions – higher highs and lower lows – because the buffering effect of marshes will no longer exist. Changes in salinity could affect predators and disease, as well as the ability of spat (larval oysters) to settle.

In response to projected changes, oyster reefs may be able to shift their distribution. However, this is dependent on several factors, including the availability of hard substrates within new areas, salinity changes, and lack of impediments. If suitable, new areas could be settled by larvae; however, the current reefs may be lost. In the Mississippi Alluvial Plain, Southern Coastal Plain, and Southern Florida Coastal Plain, experts judged coastal development to be a potential limitation to oysters’ ability to shift to new areas.

The harvesting of oyster reefs has been shown to greatly increase their vulnerability. Vertical growth on unharvested oyster reefs can keep up with projections of sea level rise, thus protecting the species themselves as well as providing continued protection against shoreline erosion [79]. In contrast, when harvested, the reefs are kept at low elevations and therefore may suffer from factors such as low dissolved oxygen and sedimentation.

Vulnerability

Oyster reefs were judged to be highly vulnerable in all subregions, except the Southern Coastal Plain, where they are moderately vulnerable. In the Southern Coastal Plain, assessors noted there was not enough information to score several of the Potential Impacts criteria that affected the average vulnerability score. Altered hydrology was judged to be the biggest threat to oyster reefs. The inability of the physical structures to migrate away from threats also increases their vulnerability.

For each subregion, the vulnerability of oyster reef was calculated by averaging the scores from the Ecosystem Status and Potential Impact modules. Scores in the Ecosystem Status and Potential Impact modules were averaged across experts.

**Eastern Oyster**

Potential Impact (Exposure + Sensitivity)

The ability of an oyster reef to keep pace with sea level rise depends on whether reef recruitment and oyster growth, minus any removal from harvest, exceed sea level rise rates. Harvested reefs should be able to keep up with moderate sea level rise, if managed sustainably. Sustainable harvesting requires taking no more shells than necessary so that substrate exists for future settlement [80].

The ranges of the projected changes in sea surface temperature (SST), salinity, and precipitation are likely to have subtle, and in many cases interactive effects on oyster recruitment, growth, and mortality [78]. Experts were less concerned with environmental conditions exceeding physiological thresholds of oysters and more concerned with the potential increase in the presence of disease and predators associated with increased salinity and SST. *Perkinsus marinus* is a protist parasite that causes the disease known as dermo or perkinsosis in eastern oysters, causing massive mortality in oyster populations. Higher temperatures and salinity are associated with major outbreaks [81]. Predation by oyster drills (*Urosalpinx cinerea*) can decrease oyster populations. Oyster drills are dormant between 10°and 12.5°C, and are generally not found below salinity of 15 [82]. Increase in SST and salinity could prolong the predators’ active period and range.

Potential increases in extreme conditions, such as increased frequency and severity of drought and flood cycles, could negatively affect oysters. Assessors noted that these impacts would be a direct result of oysters’ exposure to extreme ranges of their tolerance in temperature and salinity. Increasing drought conditions can result in hypersalinity, as has occurred in Texas, while flood cycles may increase freshwater input during spring and summer periods critical to oyster spawning [83]. If the floods reduce salinity significantly, spawning and recruitment may not occur during that year, affecting population dynamics.

Sedimentation from runoff and storm surge can smother reefs and is especially a risk to oyster reefs found in bays and enclosed areas. Runoff can also carry pollutants into estuaries and contribute to oyster mortality [39].

Adaptive Capacity

The trait most limiting to the adaptive capacity of the eastern oyster is its limited ability to disperse away from potential threats. Oysters are more limited in their ability to disperse compared to other species that are mobile throughout most of their lifetimes. Assessors noted that rapid changes in environmental conditions would be deleterious for oysters; however, oysters probably could migrate away from a gradual shift in conditions as long as hard substrate is available for larvae. Another trait that enhances their adaptive capacity is their high fecundity rate. Oysters can produce two generations per year and an estimated range of 10 – 20 million eggs per spawn [84]. Oysters can also alter shell growth patterns based on substrate, temperature, current, turbidity, and pollution [85]. The ability to shift phenotypes suggests that oysters may be able to adjust to new environmental conditions. This could be especially useful in the presence of predators. The eastern oyster responds to the presence of an oyster drill by allocating more resources toward shell growth [86]. Lastly, eastern oysters were scored as having high genetic diversity. A large gene pool increases the chances that a few individuals possess traits that will allow them to survive new conditions.

Vulnerability

Eastern oysters were judged to be moderately vulnerable across all subregions. The species assessment of eastern oysters indicates lower vulnerability than the ecosystem assessment because it takes into consideration that oyster larvae are mobile and can colonize new areas if conditions are suitable. However, because the eastern oyster is also a commercially valuable species, this vulnerability ranking can be drastically altered if oysters are harvested unsustainably [80]. Gulf-wide threats to eastern oyster include changes to the natural hydrologic regime and increased predation from oyster drills, which may benefit from high salinities.

For each subregion, the vulnerability of eastern oyster was calculated by averaging the scores from the Potential Impact (exposure + sensitivity) and Adaptive Capacity modules. Scores in the Potential Impact and Adaptive Capacity modules were averaged across experts.

**American Oystercatcher**

Potential Impact (Exposure + Sensitivity)

American oystercatcher vulnerability increases west to east in the Gulf. In Texas and Louisiana, American oystercatcher distribution is not surrounded by coastal development and natural barriers, so they should be able to move away from threats. Assessors identified storm surge as having negative impacts on American oystercatcher in all subregions; but the impact was more severe in the Southern Coastal Plain, Central Florida Coastal Plain, and Southern Florida Coastal Plain, than in the West Gulf Coastal Plain, Laguna Madre, and Mississippi Alluvial Plain. Although severity of storm surge varied, the effects on American oystercatcher were similar across the Gulf Coast. Storm surge destroys nests and erodes nesting and roosting substrate. Storm surge could be especially problematic for nesting birds if tropical storms increase in frequency or intensity or arrive earlier. Currently, tropical storms usually occur after nesting season.

Other threats to American oystercatcher include the loss of beach-nesting habitat, including man-made dredge spoil islands and oyster reef foraging areas, to erosion and sea level rise. Although there is uncertainty in how biotic interactions will change, most assessors think there will be a negative effect on American oystercatcher. Experts noted that should resources become more limited, there could be increased competition with other birds such as laughing gulls. Prey availability may also be affected. Experts commented that laughing gulls, which will likely increase with the increasing human population, are also predators of American oystercatcher nests and young.

There is a great deal of uncertainty regarding how the combined effects of climate change, sea level rise, and land use change will affect American oystercatcher, although the overall impacts are thought to be negative. Synergistic combinations of sea level rise, erosion, storm surge, and coastal development are expected to reduce the amount of habitat available for American oystercatcher and may lead to local and even regional demographic shifts and severe population declines.

Changes in precipitation, temperature, and salinity may affect American oystercatcher prey but are not expected to be direct threats to the birds.

Adaptive Capacity

The adaptive capacity of American oystercatcher varies across the Gulf; some of these differences could be due to insufficient information needed to answer some of the questions in the assessment. The entire East Coast population from New Jersey to Texas is estimated to be only about 11,000 individuals [42]. Experts noted that the entire Gulf of Mexico American oystercatcher breeding population is estimated to be 700 individuals. These birds are solitary nesters, so at the local scale extirpation could be possible. Their generation time is about 10 years, and they produce 2 – 3 eggs. Typically, only one chick will fledge.

The species possesses the ability to disperse away from threats. However, they exhibit high nest and roosting site fidelity, so they may not adjust as well as a species that does not exhibit site fidelity. Because they are broadly distributed from the Yucatan Peninsula to Long Island, they may be able to adjust to some environmental changes.

Vulnerability

American oystercatcher was judged to have moderate vulnerability in the Laguna Madre, Western Gulf Coastal Plain, and Mississippi Alluvial Plain. In the Southern Coastal Plain, Central Florida Coastal Plain, and Southern Florida Coastal Plain, assessors judged the species to be highly vulnerable. In the three highly vulnerable subregions, increased vulnerability was due to barriers to dispersal, such as coastal development and shoreline armoring to prevent beach erosion. Gulf-wide threats include loss of nesting habitat to sea level rise and synergistic effects of climate change, sea level rise, and urbanization.

For each subregion, the vulnerability of American oystercatcher was calculated by averaging the scores from the Potential Impact (exposure + sensitivity) and Adaptive Capacity modules. Scores in the Potential Impact and Adaptive Capacity modules were averaged across experts.

**Red Drum**

Potential Impact (Exposure + Sensitivity)

Across the Gulf Coast, red drum may be moderately impacted by future environmental conditions. As adults, they spend most of their time offshore, where spawning occurs. Assessors think that open water habitat is likely to increase as a result of sea level rise. The greatest impacts are likely to occur in estuaries where larval and juvenile red drum seeks shelter in the sea grass beds and marsh edges. Red drum abundance seems to be limited by total estuarine habitat [44]. Consequently, loss of marsh and sea grass beds to sea level rise and erosion could negatively affect red drum. The lack of protected habitat is also a threat to this popular recreational fishery. Protection of young fish in bays and estuaries may restore offshore spawning stock [87]. Experts feel that projected changes in temperature, precipitation, and land use are not likely to impact the species.

Adaptive Capacity

In general, red drum characteristics will likely help them cope with projected changes. Most assessors think the fish have high genetic diversity, which may increase fitness [88]. Experts commented that geographically distinct populations exhibit different life history strategies to cope with local conditions. Therefore, as the environment changes, red drum may be able to adapt to the new conditions. Assessors expect that red drum will have the ability to disperse from threats; however, there must be available nursery habitat. Adaptive capacity scores also reflected a difference in assessor opinion regarding genetic diversity and the phenotypic plasticity of red drum.

Vulnerability

Red drum vulnerability ranges from low to moderate across the Gulf Coast. Loss of habitat to sea level rise was not as severe in low vulnerability areas as compared to areas with moderate vulnerability. In the Western Gulf Coastal Plain and Mississippi Alluvial Plain, the loss of marsh habitat may decrease the dispersal of red drum, which increases vulnerability. In the Southern Coastal Plain, the overall vulnerability score was influenced by the relatively poor adaptive capacity scores that, as previously mentioned, reflected a difference in opinion among assessors.

For each subregion, the vulnerability of red drum was calculated by averaging the scores from the Potential Impact (exposure + sensitivity) and Adaptive Capacity modules. Scores in the Potential Impact and Adaptive Capacity modules were averaged across experts.

**Barrier Islands**

Ecosystem Status

Barrier islands in the Mississippi Alluvial Plain, Southern Coastal Plain, and West Gulf Coastal Plain subregions exhibit a complex pattern of landward or lateral migration as well as submergence [89]. Overall island area has decreased, with documented losses of the Mississippi River delta plain barrier islands going back to the 1890s. Losses over similar periods of time have also been documented for the Mississippi-Alabama barrier islands [90, 91]. In all of the subregions except for the Central Florida Coastal Plain, barrier island loss is likely to continue and surpass historical loss estimates.

Potential Impact

Barrier island beaches and dunes will continue to be transformed by sea level rise in all subregions. Many factors will affect islands differently, including island sediment budgets, structural characteristics such as dune height and width, rate of local relative sea level rise, and anthropogenic influences such as beach nourishment. As a result, some islands will be submerged and fragmented, while others will be more resilient and migrate landward or otherwise persist [92].

Across the Gulf, barrier islands likely will be able to shift their distribution in some areas but will suffer some decreases in extent. The ability to keep pace with sea level rise depends on sediment availability and decreases with higher rates of sea level rise [92]. For example, in the Mississippi Alluvial Plain where relative sea level rise rates are higher than in other areas of the Gulf, entire islands could become submerged.

Assessors scored land use change as having the greatest effects on barrier islands in the West Gulf Coastal Plain and Southern Coastal Plain. In both of these subregions, 30 – 49% of the barriers islands could be affected. All of the barrier islands may suffer from increased fragmentation due to the combined impacts of sea level rise and land use change. In a natural system, if the sediment supply is sufficient, the barrier island may retreat toward the mainland (“rollover”), maintaining its subaerial profile. Humans may indirectly impede this process through interruption of the sediment supply (e.g., jetties), or undertake direct efforts to stabilize an island in place (e.g., seawalls). In contrast, rates of erosion may be lowest and barrier island stability highest in areas that are maintained through sand nourishment [91].

The uncertainty in patterns and trends in tropical storm frequency and intensity leads to uncertainty surrounding how changes in disturbance regimes will impact barrier islands. Not all elements of the barrier island will respond in the same way. As previously mentioned, changes in storm frequency and a rise in sea level will alter amounts of upper beach vs. intertidal areas differently. Lowered island elevations and increased overwash, for example, would in the short- term likely lead to increased habitat for Wilson’s plover and snowy plover, but total submergence would result in loss of all barrier island habitats. Precipitation has an impact on the vegetation cover of sand dunes. Decreased vegetation on sand dunes will impact the mobility of dunes and sedimentation at the barrier flats; changes in storm frequency will likely affect the types and spatial distribution of dune vegetation [93]. Increased precipitation in the summer could supply the freshwater ponds within the barrier island, which are important sources of water and food for terrestrial vertebrates and birds.

Vulnerability

Barrier island vulnerability is moderate in the Laguna Madre subregion and high in the remaining four subregions in which they were assessed. Barrier islands were not assessed in the Southern Florida Coastal Plain because the underlying geology, including the offshore presence of coral reefs, is significantly different than islands throughout the rest of the Gulf. Vulnerability is lower in Laguna Madre because North Padre Island is protected, eliminating development as an issue. Although South Padre Island could be developed, the extent of development will not exceed 30% of the total barrier island. While sea level rise is a threat across all subregions, in Laguna Madre the assessor thought there were no range constraints limiting the ability of the barriers to migrate.

For each subregion, the vulnerability of barrier islands was calculated by averaging the scores from the Ecosystem Status and Potential Impact modules. Scores in the Ecosystem Status and Potential Impact modules were averaged across experts.

**Black Skimmer**

Potential Impact (Exposure + Sensitivity)

The black skimmer nests on barrier beaches within all of the subregions except the Laguna Madre and West Gulf Coastal Plain. In these two subregions, assessors noted that black skimmers use natural and man-made islands within the bays to avoid ground predators that occur on the larger barrier islands. The natural beaches are already eroding in many areas, and sea level rise will compound the loss. With the exception of the Laguna Madre subregion and the barrier islands in Louisiana, coastal development is projected to further encroach on the species’ habitat. The resulting habitat fragmentation threatens black skimmer populations in the Mississippi Alluvial Plain, Southern Coastal Plain, Central Florida Coastal Plain, and Southern Florida Coastal Plain. In these areas, breeding habitat is very limited so colonies may have lower productivity.

Projected increases in temperature may not affect adult birds, but could be limiting to eggs and chicks. Small changes in precipitation are unlikely to affect the species because they can handle brief, heavy rains as long as nests do not get over-washed. Storm surge attributed to tropical storm activity could be very detrimental to black skimmer, especially during the nesting season. Complete colony failure has been observed in the Gulf Coast region.

There is a great deal of uncertainty regarding how changes in biotic interactions due to sea level rise, climate change, and land use change will affect black skimmer. Assessors think they will likely experience negative effects due to changes in prey abundance as well as increased competition with laughing gulls and brown pelicans for limited space and resources. Black skimmer may also be negatively affected by the combined effects of climate change, sea level rise, and land use change although information is limited.

Adaptive Capacity

Across the Gulf, skimmers live in populations ranging from 50 to more than 500. They are highly mobile with the ability to disperse away from threats if nesting sites are available. Although they show little phenotypic variation, they breed from New England to South America under a range of temperature and salinity regimes and may be able to cope with projected temperature and salinity changes.

Vulnerability

Black skimmer vulnerability was judged to be the highest in the Southern Coastal Plain and Southern Florida Coastal Plain due to low adaptive capacity scores in these two subregions. Across all subregions, loss of habitat to sea level rise; impacts from storm surge and runoff; synergistic effects of climate change, sea level rise, and urbanization; and changes to the natural disturbance regime were scored as main threats.

For each subregion, the vulnerability of black skimmer was calculated by averaging the scores from the Potential Impact (exposure + sensitivity) and Adaptive Capacity modules. Scores in the Potential Impact and Adaptive Capacity modules were averaged across experts.

**Kemp’s Ridley Sea Turtle**

Potential Impact (Exposure + Sensitivity)

Kemp’s ridley may be particularly vulnerable to sea level rise because about half of them nest on barrier island beaches, which as previously noted are highly vulnerable across much of the Gulf region. Assessors noted that as this nesting habitat decreases, mainland beaches could be used for nesting; however, coastal development then becomes a greater threat. During tropical storms, nests are subject to inundation and washout from high tides, increased wave action, and heavy rainfall; therefore, increases in storm or rainfall frequency or intensity were judged to negatively affect this species. Because sex determination is temperature-dependent for this species, the projected increase in temperatures could affect hatchling sex ratios. At 30oC, nests are male dominated while a temperature of 32**°**C produces 100% females [94]. Temperatures that are higher than 32**°**C may cause complete nest mortality.

More indirect effects of climate and land use change are harder to assess due to limited data. Assessors commented that crab populations are the primary prey item of Kemp’s ridley and could be negatively impacted; however, Kemp’s ridley are highly migratory, have a varied diet, and may therefore find alternate prey. Increased human disturbance on land and in the water coupled with increased disease and bacterial infections due to increased water temperature may also prove detrimental. Increased urbanization could also lead to increases in mammalian predators such as coyotes and raccoons. Assessors noted that predators are a threat in some areas, such as Little St. George Island in Florida, where, for example, loggerhead nest loss can be >80% some years due to coyote predation. Kemp’s ridley sea turtles nest in mass events called “arribadas.” This may reduce impacts from predation, provided there is a sufficient quantity of nesters, because predators cannot locate individual nests by scent [95].

Adaptive Capacity

Of the species assessed, Kemp’s ridley was judged to have the lowest adaptive capacity. They exhibit some nest site fidelity compared to other species that were assessed, so they may be less likely than other species to migrate away from threats. The entire population nests within the Gulf of Mexico, mostly along the coast of Mexico although nest sites have spread north and south of the Mexico beaches. Experts indicated that Kemp’s ridley shows low to zero phenotypic plasticity, a trait that could potentially allow them to adjust to the environmental conditions they experience [96]. Kemp’s ridley have very low genetic variation after drastically declining during the mid-1900s. Experts noted that in 1985, there were only 702 recorded nests, compared to an estimated 40,000 in one day in 1947. The age of first reproduction is estimated to be between 10 and 15 years of age, after which females produce 2.5 nests biannually with an average of 90 eggs per nest.

Vulnerability

Kemp’s ridley were only assessed in the three subregions in which they most commonly nest, although nesting in other areas of the U.S. portion of the Gulf Coast does occur. In these three subregions, Kemp’s ridley were identified as the most vulnerable species out of the eleven evaluated. Kemp’s ridley may be sensitive to habitat loss from urban development and sea level rise. Increasing temperatures could also cause shifts in sex ratios.

For each subregion, the vulnerability of Kemp’s ridley sea turtle was calculated by averaging the scores from the Potential Impact (exposure + sensitivity) and Adaptive Capacity modules. Scores in the Potential Impact and Adaptive Capacity modules were averaged across experts.

**Wilson’s Plover**

Potential Impact (Exposure + Sensitivity)

Across the Gulf, Wilson’s plovers inhabit highly erosive, sandy beaches that are already disappearing in Texas and Louisiana. Sea level rise will exacerbate the loss of this habitat. Assessors in the Laguna Madre, Southern Coastal Plain, and Southern Florida Coastal Plain judge increasing temperatures to negatively impact the species. Although the species has broad latitudinal acceptance, higher temperatures may exacerbate stresses to eggs and chicks [97]. Possible increases in storm surge and changes to the disturbance regime were judged to negatively impact Wilson’s plover during breeding season.

Changes in biotic interactions were judged by to have negative effects on the species. Experts commented that human disturbance of nesting sites will increase in many areas of the Gulf Coast as coastal development and human traffic increases. It was also noted that as beaches become more limited, there will be more competition for nesting space with other species of shorebirds and seabirds. Fiddler crabs, which are the main food source for Wilson’s plover, may decrease in availability due to increased salinity.

Adaptive Capacity

Wilson’s plover are a highly mobile, broadly distributed species. However, they occupy a very specific habitat; narrow beaches. The Gulf Coast population is estimated to contain 3,000 – 3,200 breeding pairs [51]. Information regarding genetic diversity and phenotypic plasticity is limited, but assessors noted that the species does occupy a large range and consume different prey, indicating there may be some regional variation. Wilson’s plovers produce 1 – 2 chicks per year, with the first breeding occurring in the spring following the hatch year [51]. Compared to other birds assessed, assessors think Wilson’s plover can colonize new areas with a relatively low number of birds (repeated invasions of approximately 10 birds).

Vulnerability

Wilson’s plover vulnerability was judged to be high in the Southern Coastal Plain, Central Florida Coastal Plain, and Southern Florida Coastal Plain. In the remaining subregions, vulnerability is moderate. Wilson’s plover had the highest potential impacts score in the Southern Coastal Plain, which resulted in high vulnerability. In the Central and Southern Florida Coastal Plains, high vulnerability is due to low adaptive capacity. The loss of habitat to sea level rise; impacts from storm surge and runoff; and the synergistic effects of climate change, sea level rise, urbanization, and changes to the natural disturbance regime, were scored as the main threats across all subregions.

For each subregion, the vulnerability of Wilson’s plover was calculated by averaging the scores from the potential impact (exposure + sensitivity) and adaptive capacity modules. Scores in the potential impact and adaptive capacity modules were averaged across experts.

Literature Cited for S1-S3 Files

1. McMillan C, Sherrod CL. The chilling tolerance of black mangrove, *Avicennia germinans*, from the Gulf of Mexico coast of Texas, Louisiana and Florida. Contributions in Marine Science. 1986;29:9-16.

2. Spalding MD, Kainuma M, Collins L. World Atlas of Mangroves. London, UK: Earthscan; 2010.

3. Osland MJ, Enwright NM, Day RH, Doyle TW. Winter climate change and coastal wetland foundation species: salt marshes vs. mangrove forests in the southeastern United States. Global Change Biology. 2013;19:1482-94.

4. Godoy MD, de Lacerda LD. Mangroves Response to Climate Change: A Review of Recent Findings on Mangrove Extension and Distribution. Anais da Academia Brasileira de Ciencias. 2015;87(2):651-67. Epub 2015/05/21. doi: 10.1590/0001-3765201520150055. PubMed PMID: 25993360.

5. Armitage AR, Highfield WE, Brody SD, Louchouarn P. The Contribution of Mangrove Expansion to Salt Marsh Loss on the Texas Gulf Coast. PLOS ONE. 2015;10(5):e0125404. doi: 10.1371/journal.pone.0125404.

6. Laurance WF, Dell B, Turton SM, Lawes MJ, Hutley LB, McCallum H, et al. The 10 Australian ecosystems most vulnerable to tipping points. Biological Conservation. 2011;144(5):1472-80. doi: <http://dx.doi.org/10.1016/j.biocon.2011.01.016>.

7. Alongi DM. The Impact of Climate Change on Mangrove Forests. Current Climate Change Reports. 2015;1(1):30-9. doi: 10.1007/s40641-015-0002-x.

8. Ellison JC. How South Pacific Mangroves May Respond to Predicted Climate Change and Sea-level Rise. In: Gillespie A, Burns WCG, editors. Climate Change in the South Pacific: Impacts and Responses in Australia, New Zealand, and Small Island States. Dordrecht: Springer Netherlands; 2000. p. 289-300.

9. Barbier EB, Hacker SD, Kennedy C, Koch EW, Stier AC, Silliman BR. The value of estuarine and coastal ecosystem services. Ecological Monographs. 2011;81(2):169-93. doi: 10.1890/10-1510.1.

10. Dumas JV. Roseate Spoonbill (*Platalea ajaja*). The Birds of North America Online. Poole A, editor. Ithaca, NY: Cornell Lab of Ornithology; 2000.

11. Lorenz JJ. Impacts of water management on Roseate Spoonbills and the piscine prey in teh coastal wetlands of Florida Bay. Coral Gables, FL: University of Miami; 2000.

12. Battaglia LL, Woodrey MS, Peterson MS, Dillon KS, Visser MK. Wetlands of the northern Gulf coast. In: Batzer D, Baldwin A, editors. Wtland Habitats of North America: Ecology and Conservation Concerns. Berkeley, CA: University of California Press; 2012. p. 75-88.

13. Glenn-Lewin DC, Peet RK, Velben TT. Plant Succession: Theory and Prediction. London: Chapman and Hall; 1992.

14. Álvarez Rogel J, Ortiz Silla R, Alcaraz Ariza F. Edaphic characterization and soil ionic composition influencing plant zonation in a semiarid Mediterranean salt marsh. Geoderma. 2001;99(1–2):81-98. doi: <http://dx.doi.org/10.1016/S0016-7061(00)00067-7>.

15. Lenssen JPM, Menting FBJ, van der Putten WH. Do competition and selective herbivory cause replacement of Phragmites australis by tall forbs? Aquatic Botany. 2004;78:217-32.

16. Chabreck RH. Marsh zones and vegetative types in the Louisiana coastal marshes. Baton Rouge, LA: Louisiana State University; 1970.

17. Hellmann JJ, Byers JE, Bierwagen BG, Dukes JS. Five potential consequences of climate change for invasive species. Conservation biology : the journal of the Society for Conservation Biology. 2008;22(3):534-43. Epub 2008/06/26. doi: 10.1111/j.1523-1739.2008.00951.x. PubMed PMID: 18577082.

18. Minchinton TE, Simpson JC, Bertness MD. Mechanisms of exclusion of native coastal marsh plants by an invasive grass. Journal of Ecology. 2006;94(2):342-54. doi: 10.1111/j.1365-2745.2006.01099.x.

19. Zedler JB, Kercher S. Causes and consequences of invasive plants in wetlands: opportunities, opportunists, and outcomes. Critical Reviews in Plant Sciences. 2004;25:431-52.

20. Perry CL, Mendelssohn IA. Ecosystem effects of expanding populations of Avicennia germinans in a Louisiana salt marsh. Wetlands. 2009;29(1):396-406. doi: 10.1672/08-100.1.

21. Morgan SG, Zimmer-Faust RK, Heck Jr. KL, Loren DC. Population regulation of blue crabs (Callinectes sapidus) in the northern Gulf of Mexico: postlarval supply. Marine Ecology Progress Series. 1996;133:73-88.

22. Aguilar R, Hines AH, Wolcott TG, Wolcott DL, Kramer MA, Lipcius RN. The timing and route of movement and migration of post-copulatory female blue crabs, Callinectes sapidus Rathbun, from the upper Chesapeake Bay. Journal of Experimental Marine Biology and Ecology. 2005;319(1–2):117-28. doi: <http://dx.doi.org/10.1016/j.jembe.2004.08.030>.

23. Hench JL, Forward RB, Carr SD, Rittschof D, Leuttich RA. Testing a selective tidal-stream transport model: Observations of female blue crab (Callinectes sapidus) vertical migration during the spawning season. Limnology and Oceanography. 2004;49:1857-70.

24. Perry HM, McIlwain TD. Species profiles: life histories and environmental requirements of coastal fishes and invertebrates (Gulf of Mexico) - blue crab. Washington DC: U.S. Fish and Wildlife Service, 1986 Contract No.: Report 82(11.55).

25. Dickinson GH, Rittschof D, Latanich C. Spawning biology of the blue crab, Callinectes sapidus, in North Carolina. Bulletin of Marine Science. 2006;79:273-85.

26. Darnell MZ, Rittschof D, Darnell KM, McDowell RE. Lifetime reproductive potential of female blue crabs Callinectes sapidus in North Carolina, USA. Marine Ecology Progress Series. 2009;394:153-63.

27. Graham DJ, Perry H, Biesiot P, Fulford R. Fecundity and Egg Diameter of Primiparous and Multiparous Blue Crab Callinectes sapidus (Brachyura: Portunidae) in Mississippi Waters. Journal of Crustacean Biology. 2012;32(1):49-56. doi: 10.1163/193724011X615325.

28. Eddleman WR, Conway CJ. Clapper rail (*Rallus longirostris*). Revised June 19, 2012, by S.A. Rush and K.F. Gaines. . In: Poole A, editor. The Birds of North America Online. Ithaca, NY: Cornell Lab of Ornithology; 2012.

29. Rush SA, Mordecai R, Woodrey MS, Cooper RJ. Prey and habitat influences the movement of clapper rails in Northern Gulf coast estuaries. Waterbirds. 2010;33:389-96.

30. Rush SA, Gaines KF, Eddleman WR, Conway CJ. Clapper Rail (*Rallus longirostris*). In: Poole A, editor. Birds of North America Online. Ithaca, NY: Cornell Lab of Ornithology; 2012.

31. Bielefeld RR, Brasher MG, Moorman TE, Gray PN. Mottled Duck (*Anas fulvigula*). In: Poole A, editor. The Birds of North America Online. Ithaca, NY: Cornell Lab of Ornithology; 2010.

32. Wilson BC. North American Waterfowl Management Plan, Gulf Coast Joint Venture: Mottled Duck Conservation Plan. Albuquerque, NM: North American Waterfowl Management Group, 2007.

33. Moorman AM, Moorman TE, Baldassarre GA, Richard DM. Effects of Saline Water on Growth and Survival of Mottled Duck Ducklings in Louisiana. The Journal of Wildlife Management. 1991;55(3):471-6. doi: 10.2307/3808977.

34. Lassuy DR. Species profiles: life histories and environmental requirements (Gulf of Mexico)—spotted seatrout. Washington, DC: US Fish and Wildlife Service, Division of Biological Sciences, 1983 Contract No.: FWS/OBS-82.11.4.

35. Blanchet H, Van Hoose M, McEachron L, Muller B, Warren J, Gill J, et al. The Spotted Seatrout Fishery of the Gulf of Mexico, United States: A Regional Management Plan. Gulf State Fisheries Commission, 2001.

36. Perret WS, Weaver JE, Williams RO, Johansen PL, McIlwain TD, Raulerson RC, et al. Fishery profiles of red drum and spotted seatrout. 1980 Contract No.: Report 6.

37. Brown-Peterson NJ, Warren JW. The reproductive biology of spotted seatrout, *Cynoscion nebulosus*, along the Mississippi Gulf Coast. Gulf of Mexico Science. 2001;1:61-73.

38. Beck MW, Brumbaugh RD, Airoldi L, Carranza A, Coen LD, Crawford C, et al. Oyster Reefs at Risk and Recommendations for Conservation, Restoration, and Management. BioScience. 2011;61(2):107-16. doi: 10.1525/bio.2011.61.2.5.

39. VanderKooy S. The Oyster Fishery of the Gulf of Mexico, United States: A Regional Management Plan 2012 Revision. Ocean Springs, MS: Gulf States Marine Fisheries Commission, 2012 Contract No.: 202.

40. Jordan SJ. Sedimentation and remineralization associated with biodeposition by the American oyster *Crassostrea virginica* (Gmelin). College Park, MD: University of Maryland; 1987.

41. Buroker NE. Population genetics of the American oyster Crassostrea virginica along the Atlantic coast and the Gulf of Mexico. Marine Biology. 1983;75(1):99-112. doi: 10.1007/bf00392635.

42. Brown SC, Schulte S, Harrington B, Winn B, Bart J, Howe M, et al. POPULATION SIZE AND WINTER DISTRIBUTION OF EASTERN AMERICAN OYSTERCATCHERS. Journal of Wildlife Management. 2005;69(4):1538-45. doi: 10.2193/0022-541X(2005)69[1538:PSAWDO]2.0.CO;2.

43. Powers SP, Hightower CL, Drymon JM, Johnson MW. Age composition and distribution of red drum (*Sciaenops ocellatus*) in offshore waters of the north central Gulf of Mexico: an evaluation of a stock under a federal harvest moratorium. Fisheries Bulletin. 2012;110:283-92.

44. Yokel B. A contribution to the biology and distribution of the red drum, *Sciaenops ocellata*. Miami, FL: University of Miami; 1966.

45. Davis JT. Red drum biology and life history. TX, USA: Southern Regional Aquaculture Center, Texas Agricultural Extention Service, 1990 Contract No.: SRAC No 320.

46. Del Angel D, Gibeaut J, Su L, Lupher B, Taylor E. Barrier Island Vulnerability: Data Integration and Assessment. Harte Research Institute, Corpus Christi, TX. Available from: <http://gulfcoastprairielcc.org/science/science-projects/integrating-data-for-protecting-gulf-> of-mexico-barrier-islands/2014.

47. Gochfeld M, Burger J. Black Skimmer (*Rynchops niger*). In: Poole A, editor. The Birds of North America Online. Ithaca, NY: Cornell Lab of Ornithology; 1994.

48. National Marine Fisheries Service. Bi-national recovery plan for the Kemp’s ridley sea turtle (*Lepidochelys kempii*), second revision. In: NMFS, editor. Silver Spring, MD: NMFS; 2011.

49. Pritchard PCH, Marquez R. Kemp's Ridley or Atlantic Ridley, *Lepidochelys kempii*. IUCN Monograph No2 Marine Turtle Series. 1973.

50. Plotkin PT. National Marine Fisheries Service and U. S. Fish and Wildlife Service Status Reviews for Sea Turtles Listed under the Endangered Species Act of 1973. SIlver Spring, Maryland: National Marine Fisheries Service, 1995.

51. Zdravkovic MG. Conservation plan for the Wilson's Plover (*Charadrius wilsonia*). Manomet, Massachusetts: Manomet Center for Conservation Science, 2013.

52. Corbat CA, Bergstrom PW. Wilson's Plover (*Charadrius wilsonia*). In: Poole A, editor. The Birds of North America Online. Ithaca, NY: Cornell Lab of Ornithology; 2000.

53. Bergstrom PW. Breeding biology of Wilson's Plover. Wilson Bulletin. 1988;100:25-35.

54. Raabe EA, Roy LC, McIvor CC. Tampa Bay Coastal Wetlands: Nineteenth to Twentieth Century Tidal Marsh-to-Mangrove Conversion. Estuaries and Coasts. 2012;35(5):1145-62. doi: 10.1007/s12237-012-9503-1.

55. Krauss KW, From AS, Doyle TW, Doyle TJ, Barry MJ. Sea-level rise and landscape change influence mangrove encroachment onto marsh in the Ten Thousand Islands region of Florida, USA. Journal of Coastal Conservation. 2011;15(4):629-38. doi: 10.1007/s11852-011-0153-4.

56. Cavanaugh KC, Kellner JR, Forde AJ, Gruner DS, Parker JD, Rodriguez W, et al. Poleward expansion of mangroves is a threshold response to decreased frequency of extreme cold events. Proc Natl Acad Sci U S A. 2014;111(2):723-7. Epub 2014/01/01. doi: 10.1073/pnas.1315800111. PubMed PMID: 24379379; PubMed Central PMCID: PMCPMC3896164.

57. Saintilan N, Wilson NC, Rogers K, Rajkaran A, Krauss KW. Mangrove expansion and salt marsh decline at mangrove poleward limits. Glob Chang Biol. 2014;20(1):147-57. Epub 2013/08/03. doi: 10.1111/gcb.12341. PubMed PMID: 23907934.

58. Giri CP, Long J. Mangrove reemergence in the northernmost range limit of eastern Florida. Proceedings of the National Academy of Sciences. 2014;111(15):E1447-E8. doi: 10.1073/pnas.1400687111.

59. Doyle TW, Girod GF, Books MA. Chapter 12: Modeling mangrove forest migration along the southwest coast of Florida under climate change. In: Ning ZH, Turner RE, Doyle TW, Abdollahi K, editors. Integrated Assessment of the Climate Change Impacts on the Gulf Coast Region. Baton Rouge LA: GRCCC and LSU Graphic Services; 2003. p. 211-21.

60. Krauss KW, McKee KL, Lovelock CE, Cahoon DR, Saintilan N, Reef R, et al. How mangrove forests adjust to rising sea level. New Phytologist. 2014;202(1):19-34. doi: 10.1111/nph.12605.

61. Smith AG, Smith DG, Funnell BM. Atlas of Mesozoic and Cenozoic Coastlines. 1994.

62. McKee RA, Rooth JE. Where temperate meets tropical: multi-factorial effects of elevated CO2, nitrogen enrichment, and competition on a mangrove-salt marsh community. Global Change Biology. 2008;14:971-84.

63. Osland MJ, Enwright N, Stagg CL. Freshwater availability and coastal wetland foundation species: ecological transitions along a rainfall gradient. Ecology. 2014;95(10):2789-802. doi: 10.1890/13-1269.1.

64. Strong AM, Sawicki RJ, Bancroft GT. Effects of Predator Presence on the Nesting Distribution of White-Crowned Pigeons in Florida Bay. The Wilson Bulletin. 1991;103(3):415-25.

65. Lorenz JJ, Ogden JC, Bjork RD, Powell GVN. Nesting patterns of roseate spoonbills in Florida Bay 1935-1999: implications of landscape scale anthropogenic impacts. In: Porter JW, Porter KG, editors. he Everglades, Florida Bay, and Coral Reefs of the Florida Keys: An Ecosystem Sourcebook. Boca Raton, FL: CFC Press; 2002. p. 563-606.

66. Lorenz JJ, Frezza PF. Development of hydrologic criteria for the Southern Everglades and South Dade conveyance system to benefit roseate spoonbill colonies of Northeastern Florida Bay. Final Report to the South Florida Water Management District. Tavernier, FL: Audubon Florida, Everglades Science Center, 2007.

67. McKinney ML. EXTINCTION VULNERABILITY AND SELECTIVITY:Combining Ecological and Paleontological Views. Annual Review of Ecology and Systematics. 1997;28(1):495-516. doi: doi:10.1146/annurev.ecolsys.28.1.495.

68. Stedman S, Dahl TE. Status and trends of wetlands in the coastal watersheds of the Eastern United States 1998 to 2004. Washington, DC: National Oceanic and Atmospheric Administration, National Marine Fisheries Service and U.S. Department of the Interior, Fish and Wildlife Service, 2008.

69. Palaseanu-Lovejoy M, Kranenburg CJ, Barras JA, Brock JC. Land loss due to recent hurricanes in coastal Louisiana, U.S.A. Journal of Coastal Research. 2013;(63):97-109. doi: 10.2112/SI63-009.1.

70. Guillory V, Perry HM, VanderKooy S. The blue crab fishery of the Gulf of Mexico, United States: a regional management plan. Ocean Springs, Mississippi: Gulf States Marine Fisheries Commission 2001 Contract No.: Publication Number 96.

71. Ward GH. The Blue Crab: A Survey with Application to San Antonio Bay. Austin, TX: Center for Research in Water Resources, The University of Texas at Austin, 2012 Contract No.: MMS Contract No. M09AF15300 Biological Study of San Antonio Bay.

72. Zimmerman RJ, Minello TJ, Rozas LP. Salt Marsh Linkages to Productivity of Penaeid Shrimps and Blue Crabs in the Northern Gulf of Mexico. In: Weinstein MP, Kreeger DA, editors. Concepts and Controversies in Tidal Marsh Ecology. Dordrecht: Springer Netherlands; 2000. p. 293-314.

73. Bradshaw WE, Holzapfel CM. Evolutionary Response to Rapid Climate Change. Science. 2006;312(5779):1477-8. doi: 10.1126/science.1127000.

74. Taniguchi AK, editor Effects of the salinity, temperature, and food abundance upon survival of spotted seatrout eggs and larvae. Proceedings of the Colloquium on the Biology and Management of Red Drum and Seatrout Gulf States Mar Fish Comm Rep 5: 16; 1980.

75. Gell FR, Roberts CM. Benefits beyond boundaries: the fishery effects of marine reserves. Trends in ecology & evolution. 18(9):448-55. doi: 10.1016/S0169-5347(03)00189-7.

76. Callihan JL, Cowan JH, Harbison MD. Sex Differences in Residency of Adult Spotted Seatrout in a Louisiana Estuary. Marine and Coastal Fisheries. 2013;5(1):79-92. doi: 10.1080/19425120.2013.781559.

77. Etzold DJ, Christmas JY. A Mississippi marine finfish management plan Ocean Springs, MS: Mississippi-Alabama Sea Grant Consortium, 1979 Contract No.: No. PB-80- 180383.

78. La Peyre MK, Eberline BS, Soniat TM, La Peyre JF. Differences in extreme low salinity timing and duration differentially affect eastern oyster (Crassostrea virginica) size class growth and mortality in Breton Sound, LA. Estuarine, Coastal and Shelf Science. 2013;135:146-57. doi: <http://dx.doi.org/10.1016/j.ecss.2013.10.001>.

79. Grabowski JH, Brumbaugh RD, Conrad RF, Keeler AG, Opaluch JJ, Peterson CH, et al. Economic Valuation of Ecosystem Services Provided by Oyster Reefs. BioScience. 2012;62(10):900-9. doi: 10.1525/bio.2012.62.10.10.

80. Soniat TM, Klinck JM, Powell EN, Cooper N, Abdelguerfi M, Hofmann EE, et al. A shell-neutral modeling approach yields sustainable oyster harvest estimates: a retrospective analysis of the Louisiana state primary seed grounds. Journal of Shellfish Research. 2012;31(4):1103-12. doi: 10.2983/035.031.0421.

81. Soniat TM. Epizootiology of Perkinsus marinus disease of eastern oysters in the Gulf of Mexico. Oceanographic Literature Review. 1996;43(12):1265.

82. GARTON D, STICKLE WB. EFFECTS OF SALINITY AND TEMPERATURE ON THE PREDATION RATE OF THAIS HAEMASTOMA ON CRASSOSTREA VIRGINICA SPAT. The Biological Bulletin. 1980;158(1):49-57. doi: doi:10.2307/1540757.

83. Powell EN, Klinck JM, Hofmann EE, McManus MA. Influence of water allocation and freshwater inflow on oyster production: a hydrodynamic-oyster population model for Galveston Bay, Texas, USA. Environ Manage. 2003;31(1):100-21. Epub 2002/11/26. doi: 10.1007/s00267-002-2695-6. PubMed PMID: 12447579.

84. Galstoff P. The American oyster *Crassostrea virginica*. Washington, DC: United States Government Printing Office; 1964. 480 p.

85. Palmer RE, MCarriker MR. Effects of cultural conditions on morphology of the shell of the oyster *Crassostrea virginica*. Proceedings of the National Shellfisheries Association. 1979;69:58-72.

86. Lord JP. Effect of Temperature Changes on Competitive and Predator-Prey Interactions in Coastal Epi-Benthic Communities. Mansfield, Connecticut: University of Connecticut; 2014.

87. Swingle WE. Status of the commercial and recreational fishery. In: Chamberlain GW, Miget RJ, Haby MG, editors. Manual of red drum aquaculture. College State, TX: Texas Agricultural Extension Service and Sea Grant College Program, Texas A&M University; 1987. p. 46-9.

88. Turner TF, Richardson LR, Gold JR. Temporal genetic variation of mitochondrial DNA and the female effective population size of red drum (Sciaenops ocellatus) in the northern Gulf of Mexico. Mol Ecol. 1999;8(7):1223-9. Epub 1999/08/14. PubMed PMID: 10447862.

89. Rosati JD, Stone GW. Geomorphologic Evolution of Barrier Islands along the Northern U.S. Gulf of Mexico and Implications for Engineering Design in Barrier Restoration. Journal of Coastal Research. 2009:8-22. doi: 10.2112/07-0934.1.

90. Byrnes MR, Rosati JD, Griffee SF, Berlinghoff JL. Historical Sediment Transport Pathways and Quantities for Determining an Operational Sediment Budget: Mississippi Sound Barrier Islands. Journal of Coastal Research. 2013:166-83. doi: 10.2112/si63-014.1.

91. Morton RA. Historical Changes in the Mississippi-Alabama Barrier-Island Chain and the Roles of Extreme Storms, Sea Level, and Human Activities. Journal of Coastal Research. 2008:1587-600. doi: 10.2112/07-0953.1.

92. FitzGerald DM, Fenster MS, Argow BA, Buynevich IV. Coastal Impacts Due to Sea-Level Rise. Annual Review of Earth and Planetary Sciences. 2008;36(1):601-47. doi: doi:10.1146/annurev.earth.35.031306.140139.

93. Gornish ES, Miller TE. Effects of storm frequency on dune vegetation. Global Change Biology. 2010;16(10):2668-75. doi: 10.1111/j.1365-2486.2009.02144.x.

94. LeBlanc AM, Drake KK, Williams KL, Frick MG, Wibbels T, Rostal DC. Nest temperatures and hatchling sex ratios from loggerhead turtle nests incubated under natural field conditions in Georgia, United States. Chelonian Conservation Biology. 2012;11:108-16.

95. Eckrich CE, Owens DE. Solitary versus arribada nesting in the olive ridley sea turtles (Lepidochelys olivacae): a test of the predator-satiation hypothesis. Herpetologica. 1995;51:349.

96. Fordyce JA. The evolutionary consequences of ecological interactions mediated through phenotypic plasticity. The Journal of experimental biology. 2006;209(Pt 12):2377-83. Epub 2006/05/30. doi: 10.1242/jeb.02271. PubMed PMID: 16731814.

97. Ogden JC, Baldwin JD, Bass OL, Browder JA, Cook MI, Frederick PC, et al. Waterbirds as indicators of ecosystem health in the coastal marine habitats of Southern Florida: 2. Conceptual ecological models. Ecological Indicators. 2014;44:128-47. doi: <http://dx.doi.org/10.1016/j.ecolind.2014.03.008>.
